# Proteasome inhibition overcomes resistance to targeted therapies in B-cell malignancy models and in an index patient

**DOI:** 10.1038/s41419-025-07884-7

**Published:** 2025-07-23

**Authors:** Johanne U. Hermansen, Paschalis Athanasiadis, Yanping Yin, Anne-Sofie F. Rise, Alberto J. Arribas, Luciano Cascione, Hege G. Russnes, Åslaug Helland, Anthony R. Mato, Francesco Bertoni, Geir E. Tjønnfjord, Tero Aittokallio, Sigrid S. Skånland

**Affiliations:** 1 Department of Cancer Immunology, Institute for Cancer Research, Oslo University Hospital, Oslo, Norway; 2 K. G. Jebsen Centre for B Cell Malignancies, Institute of Clinical Medicine, University of Oslo, Oslo, Norway; 3 Department of Cancer Genetics, Institute for Cancer Research, Oslo University Hospital, Oslo, Norway; 4 Oslo Centre for Biostatistics and Epidemiology (OCBE), Faculty of Medicine, University of Oslo, Oslo, Norway; 5 Institute of Oncology Research, Faculty of Biomedical Sciences, USI, Bellinzona, Switzerland; 6 SIB Swiss Institute of Bioinformatics, Lausanne, Switzerland; 7 Institute for Cancer Research, Department of Oncology, Division of Cancer Medicine, Oslo University Hospital, Oslo, Norway; 8 Institute of Clinical Medicine, University of Oslo, Oslo, Norway; 9 Department of Pathology, Oslo University Hospital, Oslo, Norway; 10 Memorial Sloan Kettering Cancer Center, New York, NY USA; 11 Oncology Institute of Southern Switzerland, Ente Ospedaliero Cantonale, Bellinzona, Switzerland; 12 Department of Haematology, Oslo University Hospital, Oslo, Norway; 13 Institute for Molecular Medicine Finland (FIMM), HiLIFE, University of Helsinki, Helsinki, Finland; 14 iCAN Precision Cancer Medicine Flagship, University of Helsinki and Helsinki University Hospital, Helsinki, Finland

**Keywords:** Chronic lymphocytic leukaemia, Non-hodgkin lymphoma

## Abstract

Treatment of B-cell malignancies with the PI3K inhibitor (PI3Ki) idelalisib often results in high toxicity and resistance, with limited treatment alternatives for relapsed/refractory patients since idelalisib is recommended as a later or last line therapy. To investigate resistance mechanisms and identify alternative treatments, we studied functional phenotypes of idelalisib-resistant B-cell malignancy models. The idelalisib-resistant KARPAS1718 model remained sensitive to Bcl-2 inhibitors (Bcl-2i), whereas the resistant VL51 model showed reduced sensitivity compared to parental cells. Sensitivity correlated with phosphorylation and expression of the Bcl-2 family members Bcl-2 and Bim. Target addiction scoring revealed high dependence on the proteasome, and proteasome inhibitors (PI) were effective across models and in primary chronic lymphocytic leukemia (CLL) cells, independently of their PI3Ki- or Bcl-2i-sensitivities. PI treatment consistently upregulated Bim and Mcl-1, while Bcl-2 increased in KARPAS1718 and CLL cells. Bcl-2i plus PI combinations led to an additive effect in these models. A multi-refractory CLL patient in the IMPRESS-Norway trial (NCT04817956) treated with Bcl-2i plus PI showed initial clinical improvement but relapsed within four months. Treatment induced Bim and Mcl-1 upregulation and reduced cytotoxic CD8^+^ T-cell and CD56^dim^ NK-cell populations. Our findings suggest that PIs may overcome resistance to targeted therapies, and warrant further studies to optimize clinical responses.

## Introduction

The phosphatidylinositol-3 kinase inhibitors (PI3Ki) idelalisib and duvelisib are approved by the European Medicines Agency (EMA) and the U.S. Food and Drug Administration (FDA) for relapsed/refractory (R/R) chronic lymphocytic leukemia (CLL), and by the EMA for R/R follicular lymphoma (FL) [1]. However, severe toxicity profiles have led to black box warnings for both idelalisib and duvelisib, and accelerated FDA approvals for idelalisib and duvelisib, as well as the PI3Ki copanlisib, have been voluntarily withdrawn due to the inability to complete or meet the endpoints of the confirmatory trials [1]. These observations have emphasized the need to reconsider clinical trial designs for targeted therapies and to have long-term follow-ups to further define adverse events [2, 3].

While the European Society for Medical Oncology (ESMO) clinical practice guidelines recommend idelalisib as a late or last line therapy [4], other treatment algorithms for R/R CLL do not consider idelalisib [5, 6]. However, the preferred therapeutic options, such as Bruton’s tyrosine kinase inhibitors (BTKi) or the Bcl-2 inhibitor (Bcl-2i) venetoclax, are not necessarily available or safe to all patients. Neither are they curative. Therefore, idelalisib still remains in clinical use [7–9], and patients who relapse on this therapy may have no remaining standard of care. Novel treatment strategies are therefore needed for idelalisib-refractory patients.

Approaches to manage idelalisib toxicity, including alternative dosing schedules and combinations, are in development [1]. A second limitation to idelalisib treatment is development of drug resistance. While resistance mechanisms to BTKi and Bcl-2i are more elucidated [10], knowledge of PI3Ki resistance mechanisms remain rather scarce [11, 12]. To improve our understanding of PI3Ki resistance mechanisms and address the need for novel therapeutic options for patients who are R/R to idelalisib, we studied idelalisib-resistant B-cell malignancy models and CLL cells from idelalisib-resistant/intolerant patients [13, 14]. Cell lines and primary CLL cells were subjected to drug sensitivity screening and (phospho)protein profiling. We identified distinct drug sensitivity phenotypes in the two in vitro models. The idelalisib-resistant KARPAS1718 model remained sensitive to Bcl-2i, while the idelalisib-resistant VL51 model showed a significant decrease in Bcl-2i sensitivity, relative to their parental counterparts. Bcl-2i sensitivity was associated with Bcl-2 transcription, expression, and phosphorylation levels in both KARPAS1718 and VL51 models.

Notably, all the models showed dependence on the proteasome, and treatment with the proteasome inhibitor (PI) ixazomib was broadly effective in both idelalisib-sensitive and -resistant lines, as well as in primary CLL cells from treatment naïve or idelalisib-resistant/intolerant patients. In a clinical setting, an R/R CLL patient who received treatment with a Bcl-2i and the PI bortezomib on the IMPRESS-Norway trial (NCT04817956) achieved an initial response with significantly improved quality of life. Our findings demonstrate that idelalisib resistance is associated with cell type-specific functional phenotypes and that proteasome inhibition may provide a broadly applicable salvage strategy for idelalisib-resistant B-cell malignancies.

## Methods

### Reagents and antibodies

Compounds for single and combination treatments are listed in Supplementary Tables 1 and 2. Antibodies and reagents for (phospho)protein profiling and immunophenotyping were previously reported [15].

### Cell lines

Idelalisib-resistant lines from VL51 [16] and KARPAS1718 [17] in vitro models were developed by prolonged exposure to the IC90 concentration of idelalisib, as described [13, 14]. APRIL/BAFF/CD40L expressing fibroblasts used for co-culture with primary CLL cells were previously described [18]. All cell lines were confirmed mycoplasma negative with the MycoAlert Detection Kit (Lonza, Basel, Switzerland).

### Patient material and ethical consideration

Buffy coats from anonymized healthy blood donors were received from the Department of Immunology and Transfusion Medicine, Oslo University Hospital, Norway. The healthy donors were age- and gender-matched, with a 3:1 ratio of men to women, and above 60 years of age. Blood samples from CLL patients were received from the Department of Haematology, Oslo University Hospital, Norway, and TG Therapeutics, New York, NY, USA (NCT02742090). Patient characteristics for the treatment naïve (*n* = 11) and idelalisib-exposed (*n* = 13) CLL patients included in the study are shown in Supplementary Table 3. All participants signed a written informed consent prior to sample collection. The NCT02742090 study was done in compliance with good clinical practice and local and national regulatory guidelines. An institutional review board at each site approved the protocol before any patients were enrolled. Research on blood samples was carried out in agreement with the Declaration of Helsinki. The study was approved by the Regional Committee for Medical and Health Research Ethics of South-East Norway (2016/947 and 28507). Isolation of B cells from healthy donors and peripheral blood mononuclear cells (PBMCs) from the CLL samples was performed as previously described [19].

### Evaluation of cell proliferation

The proliferation rate was measured by an Incucyte S3 Live-Cell analysis Instrument (Sartorius, Gottingen, Germany) for real-time assessment of cell confluence. KARPAS1718 and VL51 lines (both parental and idelalisib resistant) were seeded into a Corning 384-well-plate (cat no. 3985, Merck) with an initial density of 5000 cells/well. When indicated, the cell lines were treated with 10 μg/ml anti-human CD210 (IL-10 R) antibody (Cat no. 308802, BioLegend) or 20 ng/ml IL-10 (Cat no. H7541, Sigma-Aldrich). The cells were incubated in the Incucyte at 37 °C with 5% CO_2_. Cellular confluency from three technical replicates for each condition were acquired every three hours for 72 h, using a ×10 objective lens and analyzed by the Incucyte S3 image analysis software.

### CellTiter-Glo luminescent cell viability assay

Dose-response experiments were performed as previously described [20]. Briefly, each single compound or combination (Supplementary Tables 1, 2) were printed into four 384-well cell culture microplates at five different concentrations ranging from 1 nM to 10,000 nM (0.1 nM to 1000 nM for copanlisib and dasatinib; 1000 nM to 10,000 μM for sodium salicylate). Combinations were designed using the fixed molar concentration series identical to those used for single agents. Optimal seeding density for the parental and idelalisib-resistant VL51 and KARPAS1718 cell lines was determined by using an Incucyte S3 Live-Cell analysis Instrument (Sartorius, Gottingen, Germany) [21]. Primary CLL cells were first co-cultured with irradiated APRIL, BAFF and APRIL + CD40L expressing fibroblasts for 24 h, as described [18], then separated from the adherent fibroblast layer. A single cell suspension (5000 cells/well for the KARPAS1718 and VL51 lines and 10,000 cells/well for CLL cells) was distributed to each well of the assay plate. The cells were incubated with the compounds at 37 °C for 72 h. Cell viability was measured using the CellTiter-Glo luminescent cell viability assay (Promega, Madison, WI, USA). The response readout was normalized to the negative (0.1% DMSO) and positive (100 μM benzethonium chloride) controls. The measured dose-response data were processed with the KNIME software (KNIME AG, Zurich, Switzerland).

### Enzyme-linked immunosorbent assay (ELISA)

ELISA assays were performed according to the manufacturer protocol using the DuoSet ELISA Human IL-10 kit (Cat no. DY217B) and DuoSet Ancillary Reagent Kit 2 (Cat.no DY008B) from R&D Systems (Minneapolis, MN, USA). Briefly, collected supernatants from 2 million cells were cleared by centrifugation at 10,000 × *g* for 10 min, and stored at −80 °C until further processing. The supernatants were thawed and transferred to a pre-coated human IL-10 ELISA plate followed by incubation with reagent diluent, detection antibody, Streptavidin-HRP and lastly substrate solution. Standards and samples were run in triplicates, and absorbances at 450 nm and 550 nm were measured using a Sunrise plate reader (Tecan Treading, Switzerland). The concentrations of IL-10 were calculated by generating a four-parameter logistic curve-fit using GraphPad Prism 9 (San Diego, CA, USA).

### (Phospho)protein profiling with fluorescent cell barcoding and immunophenotyping

Experiments were performed as previously described [15]. Briefly, cells were treated or not with the indicated drug for 24 h, then stained with fixable viability stain and fixed. The cells were barcoded and combined as described [15]. The cells were then stained with surface markers as indicated in the figure legends (primary cells), permeabilized, and stored at −80 °C until further processing. The samples were next stained with antibodies against intracellular (phospho)proteins and analyzed with a BD LSR Fortessa cytometer (BD Biosciences) or a Cytek 5 L Aurora (Cytek BioSciences, Fremont, Californina, USA), as indicated in the figure legends. The data were analyzed in Cytobank (https://cellmass.cytobank.org/cytobank/), as described [15]. The raw median fluorescence intensity (MFI) data were noise corrected by subtracting the signal of the isotype control and log10 transformed when not normalized against a DMSO or an internal control. These data are presented in the main figures as graphs. For transparency, the raw data are shown in the supplement as histograms. The histograms show the uncorrected raw MFI.

### RNA sequencing data analysis

RNA sequencing data from two biological replicates of parental and idelalisib-resistant KARPAS1718 and VL51 cell lines, obtained from GSE173984 [13, 14], were reanalyzed in this work using Python (v3.7). The cell lines were analyzed separately to investigate cell line-specific differences. We implemented different filtering criteria than in the previous studies [13, 14]. For each transcript, we calculated the Counts Per Million (CPM) by dividing the count by the cell line count sum and then multiplying by a million, and Mean of Counts (MC) by taking the average value of counts between the parental and resistant cell line. To calculate the Log_2_ Fold Change (L_2_FC) for each transcript, the count data were first transformed to log_2_ values. Then, for each transcript separately, the logged count in the parental line was subtracted from the logged count of the transcript in the resistant line. Since some transcripts may have zero counts, a small value (+1) was added to all the counts to make the counts non-zero. Transcripts with CPM > 0.5, MC ≥ 10, and (|L2FC|) > 2 were considered to have an altered expression. The RNA counts of the 365 transcripts that showed differential transcription between parental and idelalisib-resistant cell lines are listed in Supplementary Table 4.

### Pathway analysis

The gene transcripts identified in the RNA sequencing analysis were analyzed in Reactome (https://reactome.org) to explore the altered pathways between the cell lines and their variants. Fold enrichment was used to quantify the over-representation of a pathway based on the input gene set.

### RNA isolation and real-time polymerase chain reaction (qPCR)

Cells were collected for RNA isolation and processed following the manufacturer’s protocol (Direct.zol RNA MiniPrep kit, Cat No. R2052, Zymo Research, Irvine, CA, USA). The RNA content was measured with NanoDrop (Thermo Fisher Scientific), and 1 µg RNA was used for cDNA synthesis following the manufacturer’s protocol (RevertAid First Strand cDNA Synthesis Kit, Cat No. K1622, Thermo Fisher Scientific). PCR amplification was carried out in triplicate using PowerTrack SYBR Green master mix (Thermo Fishier, Cat no. A46109) on the CFX Connect Real-Time system (Bio-Rad, Hercules, California, USA). β-Actin was used as the internal control. The primers for IL-10 and β-Actin were: IL-10-F: 5’-GGCACCCAGTCTGAGAACA-3’; IL-10-R: 5’-ACAAGTTGTCCAGCTGATCCT-3’; β-Actin-F: 5’-GTGAAGGTGACAGCAGTCGGTT-3’; β-Actin-R: 5’-GAAGTGGGGTGGCTTTTAGGA-3’. The quantity of IL-10 mRNA was calculated as 2^-ΔΔCt^, relative to the ΔCt value of the KARPAS1718 parental line which was normalized to β-Actin [22].

### Target addiction scoring

To identify key protein dependencies in parental and idelalisib-resistant lines, we used a computational target deconvolution pipeline that is based on drug sensitivity profiles of each line separately [23]. The pipeline combines quantitative drug-cell line responses with drug-target interaction networks among both canonical on- and potential off-targets to identify pharmaceutically actionable and cell line-specific therapeutic targets. The functional essentiality of each protein target was quantified with a target addiction score (TAS), as a measure of dependency of the cell line on the therapeutic target [24]. Formally, TAS was calculated by averaging the drug sensitivity score (DSS) of each single agent and drug combination targeting the particular protein target, as annotated in Supplementary Table 5. As an outcome, each druggable target is associated with a TAS value, which provides insight about the functional importance of the protein target for the sensitivity of the cell line to inhibition by monotherapy or drug combination. Higher TAS values indicate higher functional importance.

### Data analysis

Data were processed and visualized using GraphPad Prism 9 and Python 3.7 [25]. Applied statistical tests are indicated in the figure legends. The Shapiro–Wilk test was performed to test normality of the data. To quantify the compound responses, a modified DSS was calculated for each sample and compound separately [26]. Area under the dose-response curve was calculated using an activity window from 100% to 10%, and a dose window from the minimum concentration tested to the concentration where the viability reached 10%. DSS3 metric was used, without the division by the logarithm of the upper asymptote of the logistic curve. The score ranges between 0 and 100, and a higher DSS indicates higher sensitivity to the treatment. The DECREASE tool was used to predict the full dose-response matrix based on the diagonal (dose ratio 1:1) combination experiments [27]. Combination synergy was assessed using the Bliss model in the SynergyFinder web-tool [28]. Synergy scores were interpreted as follows: Bliss scores below −10 suggest an antagonistic interaction between the two drugs, scores from −10 to 10 indicate an additive interaction, and scores above 10 indicate a synergistic interaction.

## Results

### Idelalisib-resistant lymphoma models show altered IL-10 signaling

**Fig. 1:**
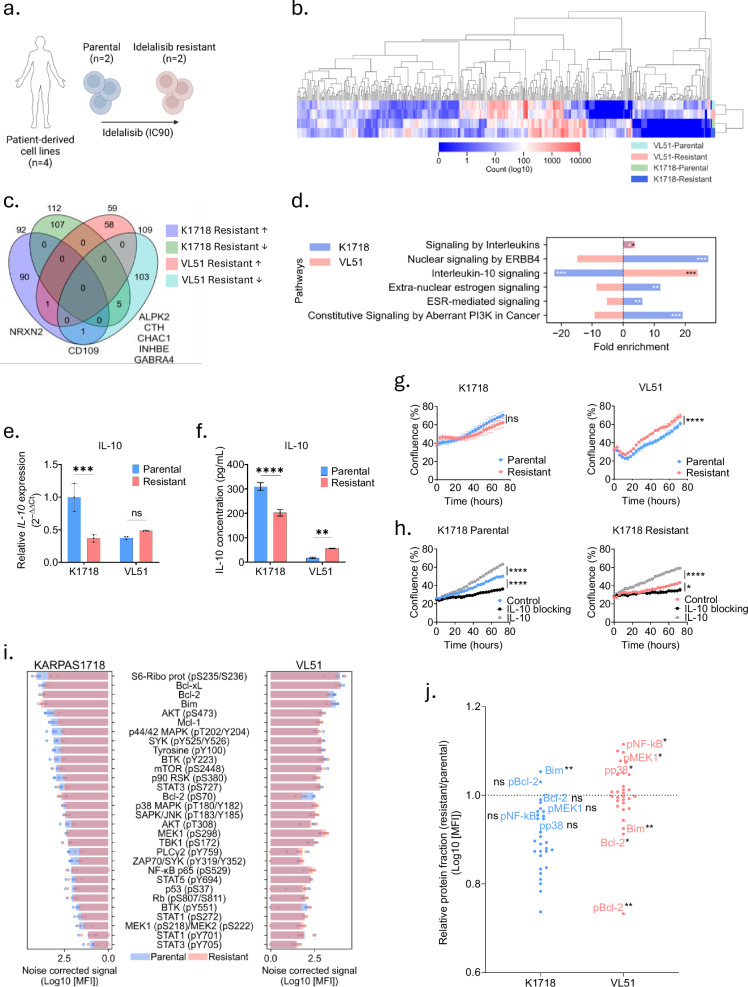
Idelalisib-resistant B-cell malignancy models show distinct functional phenotypes. **a** Illustration of the cell line models used in this study. KARPAS1718 and VL51 parental (blue) cells were made resistant to idelalisib (pink) by continuous exposure to a sub-lethal dose of idelalisib. The figure was made with biorender.com. **b** The heatmap shows the RNA counts of the 365 transcripts with differential transcription between parental and idelalisib-resistant cell lines. **c** The Venn diagram shows the overlapping transcripts between the KARPAS1718 and VL51 cell lines. The colored circles indicate up- or downregulated (arrows) transcripts in the resistant models, compared to their parental counterparts. **d** Pathway analysis of the cell lines showing the percentage of genes in a gene list belonging to the indicated pathway. A positive value of the fold enrichment indicates that the pathway is upregulated in the resistant cell line relative to the parental cell line. Blue bars refer to the KARPAS1718 model, while pink bars refer to the VL51 model. The asterisks show the FDR level of the fold enrichment of the pathways for each cell line and their variants calculated using Benjamini-Hochberg procedure. *p < 0.05, **p < 0.01, ***p < 0.001. **e** RNA was isolated from the indicated cell lines, followed by cDNA synthesis. qPCR analyses were performed with primers against IL-10 and β-actin. The barplot shows the relative quantity of IL-10 mRNA expression in the KARPAS1718 and VL51 parental (blue) and resistant (pink) models. The quantity of IL-10 (2^-ΔΔCt^) was calculated relative to the ΔCt value of KARPAS1718 parental IL-10 expression normalized to the reference gene β-actin. The plot shows the mean of three replicates, ±standard deviation (SD). Statistics were assessed with a 2-way ANOVA test with Bonferroni’s multiple comparison correction. **p < 0.01. **f** The supernatant from parental (blue) and resistant (pink) KARPAS1718 and VL51 cells were cleared by centrifugation and the quantities of human IL-10 (pg/mL) were measured using ELISA. The plot shows the mean of three replicates ±SD. Statistics were assessed with a 2-way ANOVA Bonferroni’s multiple comparison correction. ***p < 0.001, ****p < 0.0001. **g** Parental (blue) and idelalisib-resistant (pink) KARPAS1718 (left graph) and VL51 (right graph) cell lines were seeded in a 384-well plate with a start concentration of 5000 cells/well. The cell confluency was measured over time using an Incucyte S3 live cell imaging analyzer for 72 h. Growth curves of the cell lines show the confluence versus time. The plots show the mean of five replicates ±SD. Statistics were assessed with a two-way repeated measures ANOVA with Greisser-Greenhouse correction. ****p < 0.0001. **h** Parental (left graph) and idelalisib-resistant (right graph) KARPAS1718 lines were seeded in a corning 384-well plate with a start concentration of 5000 cells/well. The cells were either left untreated (control), or treated with 10 μg/ml anti-human CD210 (IL-10 R) antibody (black curve) or 20 ng/ml IL-10 (gray curve). The cell confluency was measured over time using an Incucyte S3 live cell imaging analyzer for 72 h. Growth curves of the cell lines show the confluence versus time. The plots show the mean of three replicates ±SD. Statistics were assessed with a two-way repeated measures ANOVA with Geidder-Greenhouse correction and Dunnett’s post hoc test to compare treatments to control. *p < 0.1, ****p < 0.0001. **i** Parental (blue) and idelalisib-resistant (pink) versions of KARPAS1718 (left graph) and VL51 (right graph) cell lines were fixed, permeabilized and stained with antibodies against 31 (phospho)proteins as indicated. The cells were analyzed with a BD LSR Fortessa flow cytometer, and the data were analyzed with Cytobank (https://cellmass.cytobank.org/cytobank). Raw median fluorescence intensity (MFI) data were noise corrected by subtracting the signal of an isotype control, and log10 transformed. The graphs show the mean (bars) and individual (circles) signals for *n* = 3–4 independent experiments for each cell line. **j** Experiments are described in (**i**). The relative protein fraction (resistant/parental cell line) was calculated for KARPAS1718 and VL51 cell lines. The graph shows the mean fraction from *n* = 3–4 experiments for *n* = 29 (phospho)proteins [STAT3 (pY705) and STAT6 (pY641) were excluded due to missing data]. Statistics were performed using an unpaired two-tailed parametric t-test comparing the parental and resistant cell lines. ns not significant, *p < 0.05, **p < 0.01.

To study mechanisms of idelalisib resistance, we used the parental and idelalisib-resistant versions of the lymphoma cell lines KARPAS1718 and VL51 as disease models (Fig. 1a). First, we analyzed RNA sequencing data collected from the four cell lines and found that KARPAS1718 and VL51 parental cell lines showed relatively distinct transcriptional profiles (Fig. 1b). Additional changes were induced by acquired resistance to idelalisib (Fig. 1b). Overall, 365 differentially expressed transcripts were identified in parental versus idelalisib-resistant cell lines; 204 for KARPAS1718 and 168 for VL51, with 7 transcripts overlapping between the cell lines (Fig. 1c and Supplementary Table 4). We next performed a pathway analysis based on the RNA transcriptomic changes (Fig. 1d). We confirmed previous findings that overexpression of the ERBB4 pathway is involved in idelalisib resistance in the KARPAS1718 cell line [14]. Of interest, we further observed that the IL-10 signaling pathway was upregulated in the VL51 resistant cell line, while downregulated in the KARPAS1718 resistant cell line, compared to parental lines (Fig. 1d). To validate these findings, we performed qPCR and ELISA to quantify the gene expression and secretion of IL-10 (Fig. 1e, f). In agreement with the RNA sequencing data, we observed that IL-10 gene expression was significantly lower in the resistant KARPAS1718 line compared to the parental line, and higher, although not statistically significantly, in the resistant VL51 cell line compared to its parental counterpart (Fig. 1e). Furthermore, we observed that the secreted IL-10 level was significantly lower in the resistant KARPAS1718 cell line, and significantly higher in the resistant VL51 cell line when compared to their parental counterparts (Fig. 1f). It has been reported that IL-10 can contribute to growth of B cells [29], and by studying the growth curves of the cell lines in a live imaging assay, we confirmed that the resistant VL51 cell line proliferated statistically faster than the parental line, while the opposite trend was observed for the resistant KARPAS1718 model, although this difference was not statistically significant (Fig. 1g). To confirm that the growth was dependent on IL-10, we treated KARPAS1718 cells with an IL-10 receptor antibody to block IL-10 binding, or with soluble IL-10. We observed that the growth was inhibited by the blocking of IL-10 and stimulated by IL-10 treatment, both in the parental and resistant lines (Fig. 1h). The same treatment responses were observed in the VL51 parental and resistant lines as well (data not shown). These findings confirm that the cell proliferation depends on IL-10 signaling.

Taken together, we show that the two idelalisib-resistant models have different transcriptional phenotypes, and that idelalisib resistance induces changes in cellular pathways related to cell growth and resistance to apoptosis.

### Idelalisib-resistant VL51 cells, but not KARPAS1718, show reduced expression of Bcl-2 family proteins

To investigate how the difference in RNA transcript profiles affected cell signaling, we performed (phospho)protein profiling of the four cell lines (Fig. 1i, Supplementary Fig. 1a). Overall, we observed that (phospho)protein levels were slightly reduced in idelalisib-resistant KARPAS1718 cells, but not in idelalisib-resistant VL51 cells, compared to parental lines (Fig. 1i, j). Of interest, we found that the Bcl-2 family proteins Bcl-2, Bcl-2 (pS70), and Bim differed from this trend, as they were significantly reduced (*p* < 0.05) in idelalisib-resistant VL51 cells, but not in idelalisib-resistant KARPAS1718 cells, compared to parental cells (Fig. 1i, j). Consistent with this, we also observed that *BCL2* transcripts were reduced in the idelalisib-resistant VL51 cells relative to the three other lines (Supplementary Fig. 1b). Together, these findings suggest that idelalisib resistance may affect apoptotic pathways.

We also observed a significantly increased phosphorylation of NF-κB p65 (pS529) in idelalisib-resistant VL51 cells, while this was reduced in idelalisib-resistant KARPAS1718 cells, relative to parental lines (Fig. 1j). NF-κB activation is reported to contribute to IL-10 production [30], and this finding therefore aligns with the observed increase in IL-10 secretion in VL51 cells upon idelalisib resistance (Fig. 1f). Together, our findings reveal distinct functional phenotypes in the two idelalisib-resistant models, including varied expression of Bcl-2 family proteins and changed signaling of proteins in the IL-10 pathway.

### Idelalisib-resistant VL51 cells, but not KARPAS1718, show reduced sensitivity to Bcl-2i

**Fig. 2:**
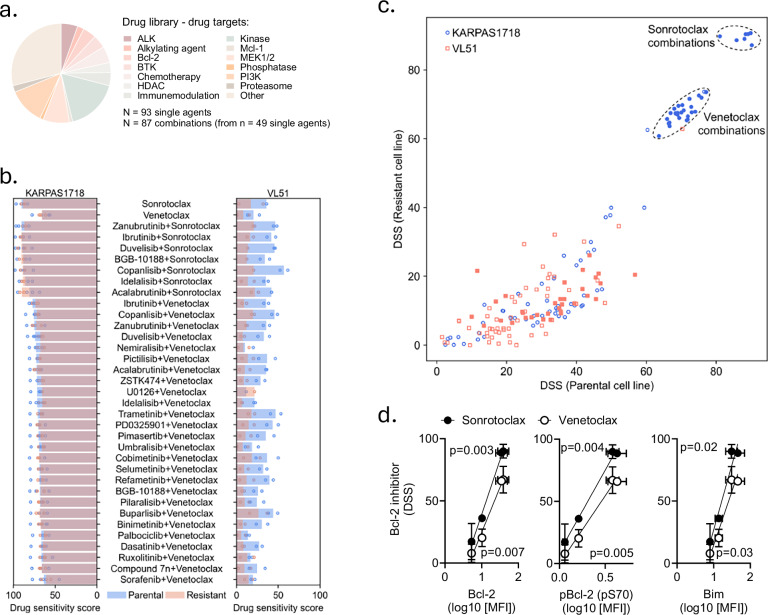
Bcl-2 and Bim phosphorylation/expression correlates with sensitivity to Bcl-2i. **a** Illustration of the drug library and therapeutic targets. **b** Parental (blue) and idelalisib-resistant (pink) KARPAS1718 (left graph) and VL51 (right graph) cell lines were treated with drugs or drug combinations at five different concentrations (1–10,000 nM) for 72 h. Cell viability was then assessed with the CellTiter-Glo luminescent cell viability assay. The graphs show the mean (bars) and individual (circles) drug sensitivity scores (DSS) to the different treatments for *n* = 2–3 independent experiments for each cell line. The scores were calculated based on the area under the dose-response curves. High score indicates high sensitivity to the treatment. **c** Parental (x-axis) and idelalisib-resistant (y-axis) KARPAS1718 (blue circles) and VL51 (pink squares) cell lines were treated with 87 drug combinations at five different concentrations (1–10,000 nM) for 72 h. Cell viability was then assessed with the CellTiter-Glo luminescent cell viability assay. The plot shows the mean DSS to the different treatments for *n* = 2–3 independent experiments for each cell line. The scores were calculated based on the area under the dose-response curves. Filled symbols indicate drug combinations that include the Bcl-2i sonrotoclax or venetoclax. **d** Pearson correlation analysis was performed on the mean (phospho)protein levels detected in Fig. 1h and the drug sensitivity score (DSS) to sonrotoclax (black circles) and venetoclax (white circles) described in (**b**), where the points indicate the parental and idelalisib-resistant versions of KARPAS1718 and VL51 cell lines. The *p*-values are shown for each correlation (two-tailed Pearson r). The error bars indicate standard deviation (SD).

To study the impact of the phenotypic changes induced by idelalisib resistance in KARPAS1718 and VL51 cells on drug sensitivity profiles, we next performed a drug sensitivity screen on the four lines using a drug library consisting of 93 single agents (Fig. 2a and Supplementary Table 1) and 87 drug combinations (Supplementary Table 2). The drug sensitivity score, a metric based on the area under the drug dose-response curve [26], was calculated for each single agent and cell line (Supplementary Fig. 2a). Reduced sensitivity to idelalisib in the resistant strains was confirmed as previously demonstrated [14, 20] (Supplementary Fig. 2b). Here, we found that the sensitivity to several drugs, including the PI3Ki copanlisib, the mTOR inhibitor everolimus, and the BTKi zanubrutinib, was markedly reduced upon idelalisib resistance in both models (Supplementary Fig. 2a). Of interest, we found that both parental and idelalisib-resistant KARPAS1718 cells were highly sensitive to Bcl-2 inhibition, either with sonrotoclax (DSS ± SD = 90 ± 5,5 and 89 ± 2,4, respectively) or venetoclax (DSS ± SD = 65 ± 8,7 and 65 ± 5,2, respectively) (Fig. 2b, left); while the sensitivity to these drugs was much lower in the VL51 parental cells [DSS = 36 (*n* = 1) for sonrotoclax and DSS ± SD = 20 ± 9,9 for venetoclax] (Fig. 2b, right). This sensitivity was reduced further in the idelalisib-resistant VL51 strain (DSS ± SD = 17 ± 20 for sonrotoclax and 7 ± 10 for venetoclax) (Fig. 2b, right), confirming distinct functional phenotypes in the two idelalisib-resistant models.

When studying the effects of combination treatments (Supplementary Fig. 2c), we found that the sensitivity to combinations that included a Bcl-2 inhibitor (sonrotoclax or venetoclax, *n* = 33) showed a similar pattern with a sustained and higher sensitivity in KARPAS1718 parental and idelalisib-resistant lines and lower sensitivity in VL51 resistant cells, relative to the parental cells (Fig. 2b). Indeed, the Bcl-2 inhibitor combinations were the most effective combinations in KARPAS1718 parental and idelalisib-resistant cell lines (Fig. 2c). Sonrotoclax combinations were more effective than venetoclax combinations (Fig. 2c), which aligns with its higher efficacy as a single agent (Fig. 2b). Sensitivity to most other tested combinations was markedly reduced in both KARPAS1718 and VL51 idelalisib-resistant cells, when compared to their parental counterparts (Supplementary Fig. 2c).

It has been reported that durable response to venetoclax is associated with high expression of Bcl-2 and/or Bim in acute myeloid leukemia (AML) [31], and Bcl-2 family (phospho)protein levels have been shown to associate with sensitivity to venetoclax in CLL, multiple myeloma (MM), and mantle cell lymphoma (MCL) [32–34]. We therefore investigated the association between Bcl-2 and Bim phosphorylation/expression and Bcl-2i sensitivity in our models. A correlation analysis of the (phospho)protein levels and venetoclax or sonrotoclax sensitivity revealed statistically significant correlations with all three (phospho)proteins (Fig. 2d). Of note, this correlation should be interpreted with caution due to the small sample size.

### Idelalisib-resistant KARPAS1718 and VL51 cells remain sensitive to proteasome inhibition

**Fig. 3:**
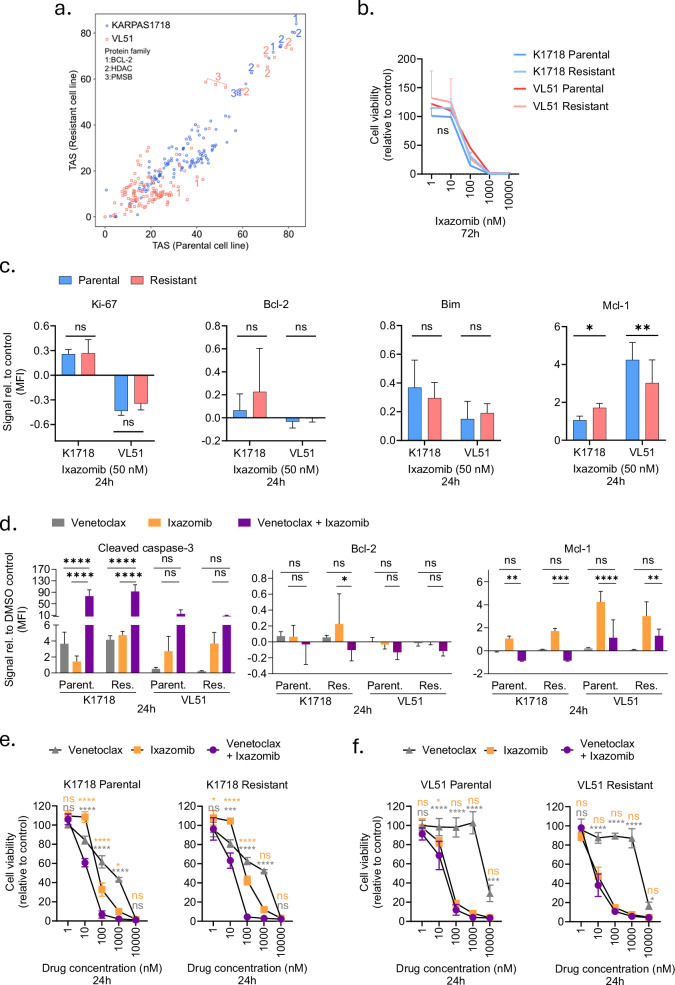
Proteasome inhibitors are effective in idelalisib-resistant B-cell malignancy models. **a** Target addiction score (TAS) of proteins in parental (x-axis) and idelalisib-resistant (y-axis) cell lines are shown. Each point indicates a single protein target. Targets with the highest addiction scores are annotated; B cell lymphoma 2 (BCL-2), histone deacetylase (HDAC), proteasome subunit beta (PMSB). **b** Parental (blue) and idelalisib-resistant (pink) versions of KARPAS1718 and VL51 cell lines were treated with ixazomib at five different concentrations (1–10,000 nM) for 72 h. Cell viability was then assessed with the CellTiter-Glo luminescent cell viability assay. The graph shows the mean cell viability for *n* = 1–3 experiments ±SD. ns not significant with a 2-way repeated measures ANOVA and Bonferroni’s multiple comparison correction. **c** Parental (blue) and idelalisib-resistant (pink) versions of KARPAS1718 and VL51 cell lines were treated with 50 nM ixazomib for 24 h. The cells were then fixed, barcoded, and permeabilized, before staining with antibodies against the indicated intracellular proteins. The cells were analyzed with a BD LSR Fortessa flow cytometer, and the data were analyzed with Cytobank (https://cellmass.cytobank.org/cytobank). The signal is shown as noise corrected median fluorescence intensity (MFI) relative to the DMSO control which was set to zero (mean ± SD, *n* = 3). Statistics were performed with a 2-way ANOVA Bonferroni’s multiple comparison correction. ns; not significant, *p < 0.05, **p < 0.01. **d** The indicated cell lines were treated with 50 nM venetoclax, 50 nM ixazomib, or 50 nM venetoclax + ixazomib for 24 h and further processed as described in (**c**). The bars show mean with standard deviation (SD) of *n* = 3 experiments. Statistics were performed with a 2-way ANOVA Bonferroni’s multiple comparison correction. ns; not significant, *p < 0.05, **p < 0.01, ***p < 0.001, ****p < 0.0001. **e** Parental (left) and idelalisib-resistant (right) versions of the KARPAS1718 cell lines were treated with venetoclax (gray), ixazomib (orange), or the combination (purple) at five different concentrations (1–10,000 nM) for 24 h in triplicates. Cell viability was then assessed with the CellTiter-Glo luminescent cell viability assay. The graphs show the mean cell viability ± SD, *n* = 3. Statistics were performed with a 2-way ANOVA and Bonferroni’s multiple comparison correction when comparing single treatment to combination treatment and indicated for the comparison with venetoclax in gray and for ixazomib in orange. ns not significant, *p < 0.05, ***p < 0.001, ****p < 0.0001. **f** Parental (left) and idelalisib-resistant (right) versions of the VL51 cell lines were treated with venetoclax, ixazomib, or the combination at five different concentrations (1–10,000 nM) for 24 h as described in (**e**). The graphs show the mean cell viability ±SD, *n* = 3. Statistics were performed with a 2-way ANOVA and Bonferroni’s multiple comparison correction when comparing single treatment to combination treatment and indicated for the comparison with venetoclax in gray and for ixazomib in orange. ns not significant, *p < 0.05, ***p < 0.001, ****p < 0.0001.

To identify effective drugs or combinations in the idelalisib-resistant VL51 cells, which were less responsive to Bcl-2i, we performed a target addiction scoring based on drug sensitivity to the 93 single agents and 87 drug combinations, separately in the parental and idelalisib-resistant KARPAS1718 and VL51 models (Supplementary Table 2). Bcl-2, histone deacetylase (HDAC), and the proteasome were identified as the top three target addictions across the four lines (Fig. 3a), indicating that all the cell lines were most vulnerable to inhibition of these proteins. Proteasome inhibitors (PIs) are reported to induce cell death in several human cancers [35], and PIs are approved for the treatment of MM and MCL [36]. RNA sequencing profiles of the cell lines confirmed that RNA transcripts for the proteasome subunits and interactome remained unaffected by induced resistance to idelalisib, both in KARPAS1718 and VL51 cells (Supplementary Fig. 3a, b). We therefore tested the efficacy of proteasome inhibition on the four cell lines. Treatment with ixazomib, a next-generation, oral PI, equally reduced cell viability in both parental and idelalisib-resistant cell lines (Fig. 3b), suggesting that it can overcome resistance to targeted therapies.

To investigate the mechanisms underlying the efficacy of PIs in KARPAS1718 and VL51 cells, we performed (phospho)protein profiling of the four lines before and after treatment with ixazomib (Supplementary Fig. 4a, b). We observed an increased expression of the proliferation marker Ki-67 and the anti-apoptotic Bcl-2 protein in response to ixazomib treatment in KARPAS1718, while Ki-67 was reduced and Bcl-2 was unchanged in response to ixazomib treatment in VL51 (Fig. 3c, left and middle). Of interest, expression of the pro-apoptotic protein Bim and the anti-apoptotic protein Mcl-1 increased in response to ixazomib treatment in all four lines (Fig. 3c, right). This suggests that proteasome inhibition induces apoptosis via regulation of Bcl-2 family proteins, but the mechanism may vary between cell types. Furthermore, these findings demonstrate that proteasome inhibition can overcome both PI3Ki and Bcl-2i resistance in lymphoma models.

### PI plus venetoclax combination has an additive effect in KARPAS1718 cells, but not in VL51

Since we found that sensitivity to venetoclax correlates with Bcl-2 expression (Fig. 2d), and ixazomib treatment induces expression of Bcl-2 in KARPAS1718 cells (Fig. 3c), we next investigated whether combined PI plus venetoclax treatment has an additive effect in this model. Cells were treated with the single agents or combination for 24 h, and cleaved caspase-3 was then detected by flow cytometry as a measure of apoptosis. We confirmed that KARPAS1718 cells were sensitive to both ixazomib and venetoclax single agents, and observed a statistically significant additive effect with the combination, both in the parental and idelalisib-resistant lines (Fig. 3d, left). A similar trend was observed in the VL51 lines, but the combination effect was less pronounced and not statistically significant relative to the single agents (Fig. 3d, left). We next assessed the effect of combination treatment on Bcl-2 expression. For all four cell lines, a small reduction was observed relative to untreated cells (Fig. 3d, middle), and the increased Bcl-2 and Mcl-1 expression resulting from ixazomib treatment of KARPAS1718 cells could be reversed by the combination (Fig. 3d, middle and right).

We next tested if the observed treatment effects could be confirmed with the CellTiter-Glo viability assay. In agreement with the cleaved caspase-3 assay, we observed an additive effect of the combination in KARPAS1718 cells, but not in VL51 (Fig. 3e, f). Together, these findings suggest that proteasome and Bcl-2 inhibition may act additively in some B-cell lymphoma models.

### CLL cells from idelalisib-resistant/intolerant patients remain sensitive to PI

**Fig. 4:**
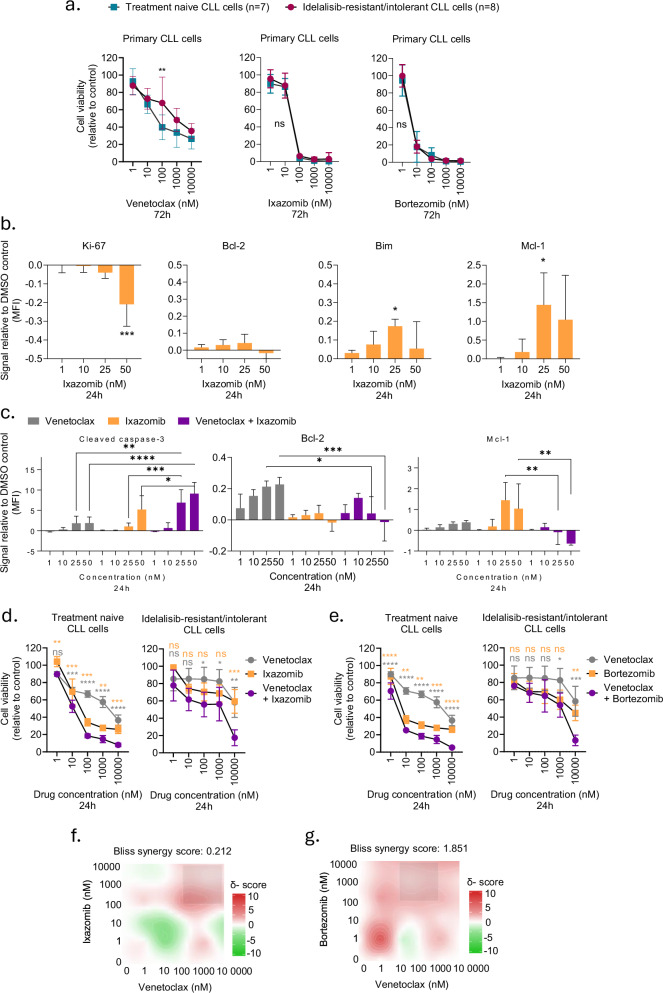
Proteasome inhibitors are effective in idelalisib-resistant primary CLL cells. **a** Peripheral blood mononuclear cells (PBMCs) from treatment naïve (*n* = 7) or idelalisib-resistant/intolerant (*n* = 8) chronic lymphocytic leukemia (CLL) patients were co-cultured with APRIL/BAFF/CD40L fibroblasts for 24 h to prevent spontaneous apoptosis of the CLL cells. The CLL cells were then separated from the fibroblast layer and treated with venetoclax (left), ixazomib (middle) or bortezomib (right) at five different concentrations (1–10,000 nM) for 72 h. Cell viability was assessed with the CellTiter-Glo luminescent cell viability assay. The cell viability was normalized to negative (0.1% DMSO) and positive (100 μM benzethonium chloride) controls. The graphs show the mean viability, and the error bars indicate standard deviation (SD). ns not significant, **p < 0.01 with a 2-way ANOVA and Bonferroni’s multiple comparison correction. **b** Freshly thawed PBMCs from treatment naïve (*n* = 4) CLL patients were treated with 0.1% DMSO or 1, 10, 25 or 50 nM ixazomib for 24 h. The cells were stained with a fixable viability stain, fixed, barcoded, and stained with anti-CD3 and anti-CD19 surface markers prior to permeabilization. The cells were then stained with antibodies against the indicated intracellular proteins. The samples were analyzed with a BD LSR Fortessa flow cytometer, and the data were analyzed with Cytobank (https://cellmass.cytobank.org/cytobank). The signal is shown for as noise corrected median fluorescence intensity (MFI) relative to the DMSO control which was set to zero (mean ± SD, *n* = 3) for CD3^−^CD19^+^ CLL cells. Statistics were performed with a one-way ANOVA test and Bonferroni corrected for multiple comparisons against the DMSO control. *p < 0.05, ***p < 0.001. **c** Experiment as described in (**b**), and the cells were treated with 0.1% DMSO or venetoclax, ixazomib or venetoclax + ixazomib at four different concentrations (1–50 nM) for 24 h. The graphs show the noise corrected median fluorescence intensity (MFI) signal relative to the DMSO control which was set to zero (mean ± SD, *n* = 3) for CD3^−^CD19^+^ CLL cells. Statistics were performed with a 2-way ANOVA test with Bonferroni correction for multiple comparisons. *p < 0.05, **p < 0.01, ***p < 0.001, ****p < 0.0001. **d** PBMCs from *n* = 4 treatment naïve CLL patients (left panel) and from *n* = 3 idelalisib-resistant/intolerant CLL patients (right panel) were co-cultured with APRIL/BAFF/CD40L fibroblasts for 24 h to prevent spontaneous apoptosis of the CLL cells. The cells were then separated from the fibroblast layer and treated with 0.1% DMSO or venetoclax (gray), ixazomib (orange) or venetoclax + ixazomib (purple) at five different concentrations (1–10,000 nM) for 24 h in triplicates. Cell viability was assessed with the CellTiter-Glo luminescent cell viability assay. The cell viability was normalized to the negative (0.1% DMSO) control. The graphs show the mean viability of triplicates, and the error bars indicate standard deviation (SD). Statistics were performed with a 2-way ANOVA with Bonferroni correction when comparing single treatment to combination treatment and indicated for the comparison with venetoclax in gray and ixazomib in orange. ns; not significant, **p < 0.01, ***p < 0.001, ****p < 0.0001. **e** Experiments were performed as described in (**d**), but with bortezomib instead of ixazomib. **f** The data from experiment described in **d** were used in the DECREASE machine learning model (https://decrease.fimm.fi/) for predicting the full drug combination dose-response matrix, and further analyzed in SynergyFinder (https://synergyfinder.fimm.fi/) for scoring the synergy of the drug combination ixazomib and venetoclax. Average Bliss synergy score of the full matrix is indicated. **g** As described in (**f**), but with data from the experiments performed in (**e**).

To test whether our findings on cell line models could be confirmed in patient cells, we studied PBMCs from treatment naïve or idelalisib-resistant/intolerant CLL patients. This model was chosen since idelalisib is an approved therapy for R/R CLL [1]. We have previously shown that the sensitivity to idelalisib is reduced in CLL cells from the idelalisib-resistant/intolerant patients relative to cells from the treatment naïve patients [20]. Similarly to what was observed in the VL51 model, we found that Bcl-2 phosphorylation and expression levels were significantly reduced in CLL cells from idelalisib-resistant/intolerant patients relative to treatment naïve patients (Supplementary Fig. 5a). However, Bim levels were comparable at the two conditions (Supplementary Fig. 5a). We further observed that sensitivity to Bcl-2 inhibitors (venetoclax and sonrotoclax), both as single agents and in combinations, was reduced in CLL cells from idelalisib-resistant/intolerant patients compared to CLL cells from treatment naïve patients (Fig. 4a, left, and Supplementary Fig. 5b). This is in agreement with the observed reduction in Bcl-2 expression, and most closely resembles the phenotype of the VL51 model. We next studied the effect of PI on idelalisib-resistant CLL cells. In agreement with the findings on the cell line models, we observed that proteasome inhibition with either ixazomib or bortezomib was effective in primary CLL cells from both treatment naïve and idelalisib-resistant/intolerant patients (Fig. 4a, middle and right).

Next, we performed (phospho)protein profiling of 34 proteins on PBMCs from treatment naïve CLL patients after treatment with ixazomib (Supplementary Fig. 4c). We observed decreased expression of the proliferation marker Ki-67 in response to all tested concentrations of ixazomib (Fig. 4b, left), similarly to what was observed in VL51 (Fig. 3c). In contrast, we observed a modest increase in expression of Bcl-2 (Fig. 4b, middle), which was also observed in KARPAS1718 cells (Fig. 3c). Consistent with both lymphoma models, the expression of Bim and Mcl-1 increased in response to ixazomib treatment of primary CLL cells (Fig. 4b, right). Together, these findings suggest that Ki-67 and Bcl-2 are regulated differently, while Bim and Mcl-1 are regulated similarly by PI across different cell types.

We further studied potential additive effects of combining venetoclax and ixazomib in CLL. PBMCs were treated with the different drugs for 24 h. The shorter treatment time was chosen to increase the resolution of the drug responses since both venetoclax and PI are potent in primary CLL cells. Both single agent and combination treatment induced cleavage of caspase- 3 (Fig. 4c, left). Interestingly, the combination showed an additive effect compared to the single agents, and was statistically significantly different from single agent treatment at specific concentration points (Fig. 4c, left). As observed for the KARPAS1718 lines, Bcl-2 expression was induced in primary CLL cells by venetoclax or ixazomib single agent treatment, and most significantly by venetoclax (Fig. 4c, middle). Bcl-2 expression was significantly reduced by combination treatment when compared to venetoclax single agent, but the expression remained higher than the level induced by ixazomib single agent treatment (Fig. 4c, middle). This suggests that Bcl-2 is regulated differently by ixazomib across models. In addition, we observed that the Mcl-1 expression was significantly reduced when ixazomib was combined with venetoclax, similarly to what was observed for the lymphoma models (Fig. 4c, right).

Next, we performed CellTiter-Glo viability assays to test the effects of combination treatment on cell viability. In agreement with the cleaved caspase-3 assay, we observed a combination effect of ixazomib plus venetoclax in CLL cells from treatment naïve patients (Fig. 4d, left). A similar trend was observed for CLL cells from idelalisib-resistant/intolerant patients, but the overall sensitivity to treatments was lower than in treatment naïve CLL cells (Fig. 4d, right). These findings were confirmed with bortezomib plus venetoclax treatment (Fig. 4e). A Bliss synergy analysis showed that PI plus venetoclax combinations led to an additive effect in primary CLL cells at given drug concentrations (Fig. 4f, g, red areas), in agreement with previous reports [37, 38].

### Treatment of an R/R CLL index patient with bortezomib plus venetoclax

**Fig. 5:**
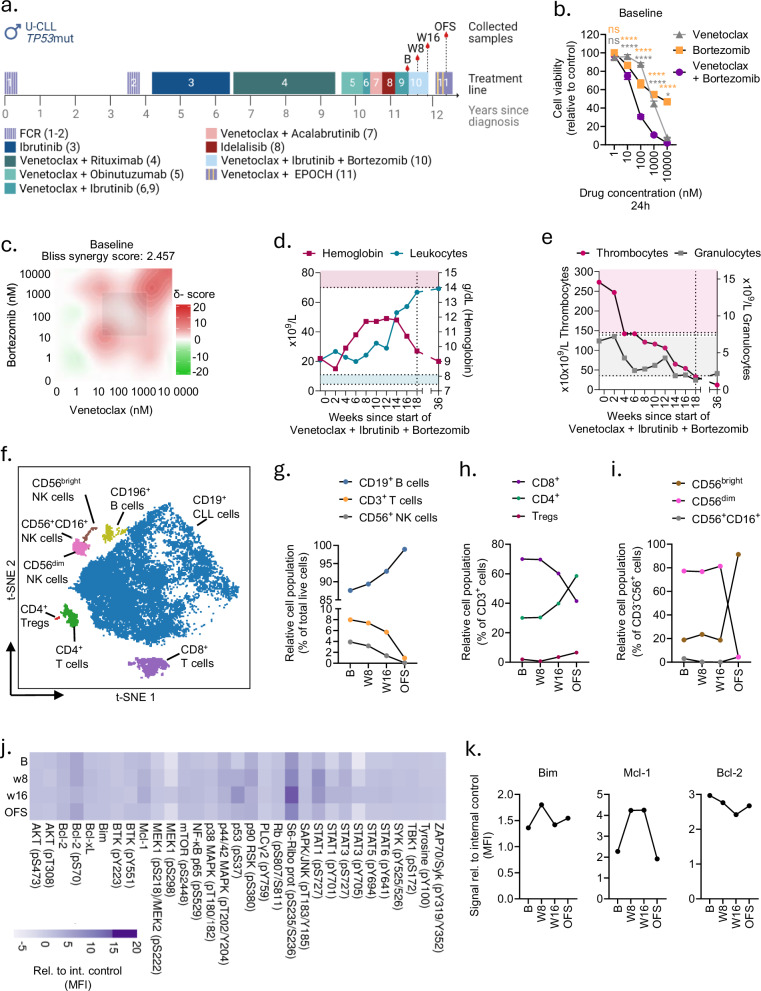
Proteasome inhibitors are effective in a multi-resistant CLL index patient. **a** Timeline illustrating the treatment history of the CLL patient. Baseline (B), week 8 (W8), week 16 (W16) and off-study (OFS) indicates the time-points when blood samples were collected from the patient. FCR fludarabine, cyclophosphamide, rituximab, EPOCH etoposide, prednisolone, vincristine, cyclophosphamide, doxorubicin. The figure was created with BioRender.com. **b** Peripheral blood mononuclear cells (PBMCs) from the baseline sample were co-cultured with APRIL/BAFF/CD40L fibroblasts for 24 h to prevent spontaneous apoptosis of the CLL cells. The cells were then separated from the fibroblast layer and treated with venetoclax (gray), bortezomib (orange), or venetoclax + bortezomib (purple) at five different concentrations (1–10,000 nM) for 24 h. Cell viability was assessed with the CellTiter-Glo luminescent cell viability assay and normalized to negative (0.1% DMSO) control. The graph shows the mean viability of triplicates, and the error bars indicate standard deviation (SD). Statistics were performed with a 2-way ANOVA with Bonferroni’s multiple comparison correction when comparing single treatment to combination treatment and indicated for the comparison with venetoclax in gray and for ixazomib in orange. ns not significant, *p < 0.05, ****p < 0.0001. **c** Data from experiment described in (**b**) were used in DECREASE (https://decrease.fimm.fi/) for predicting the full drug combination dose-response matrix, and further analyzed in SynergyFinder (https://synergyfinder.fimm.fi/) for scoring the synergy of the drug combination. Average Bliss synergy of the full matrix is indicated. **d** Hemoglobin and leukocyte counts of the CLL patient over time (in weeks) since start of venetoclax + ibrutinib + bortezomib treatment. Normal reference values are indicated with the filled areas in red and blue, respectively. **e** Thrombocyte and granulocyte counts of the CLL patient over time (in weeks) since start of venetoclax + ibrutinib + bortezomib treatment. Normal reference values are indicated with the filled areas in red and gray, respectively. **f** PBMCs collected at baseline, w8, w16 and off-study were fixed, barcoded, stained with surface markers, then permeabilized and stained with antibodies against intracellular (phospho)proteins. The samples were analyzed using a Cytek 5 L Aurora instrument, and the data were analyzed with Cytobank (https://cellmass.cytobank.org/cytobank). All live cells from the sample collected at baseline were visualized in a two-dimensional t-SNE (t-distributed stochastic Neighbour Embedding) plot generated based on the expression of the indicated surface markers. **g** Data from the experiment described in (**f**) were used to plot the relative population of CD19^+^ CLL cells (blue), CD3^+^ T-cells (orange) and CD56^+^ NK cells (gray) as percent of total live cells. **h** Data from the experiment described in (**f**) were used to plot the proportion of CD8^+^ (purple), CD4^+^ (green) and T regulatory cells (red) as percent of CD3^+^ cells. **i** Data from the experiment described in (**f**) were used to plot the proportion of NK subsets; CD56^bright^ (brown), CD56^dim^ (pink), CD56^+^CD16^+^ (gray) cells as percent of all CD3^-^ CD56^+^ cells. **j** Data from the experiment described in (**f**). Signals from CD3^−^CD19^+^ cells were selected for 31 (phospho)proteins as indicated. The raw median fluorescence intensity (MFI) data were noise corrected by subtracting the signal of an isotype control and normalized against an internal control. The heatmap was created using ClustVis (https://biit.cs.us.ee/clustvis). **k** Selected data for Bim, Mcl-1 and Bcl-2 as described in (**j**).

An R/R CLL patient with *TP53* mutation and unmutated IGHV and who had no remaining standard of care was screened for inclusion in the clinical trial IMPRESS-Norway (NCT04817956) (Fig. 5a). The patient had received nine previous treatment lines with FCR (fludarabine, cyclophosphamide, rituximab), ibrutinib, venetoclax in combination with anti-CD20 monoclonal antibodies, and idelalisib (Fig. 5a). The IMPRESS-Norway trial is a prospective, non-randomized clinical trial evaluating the efficacy of anti-cancer drugs on new indications [39]. Patients enrolled in the CLL cohort can be treated with bortezomib following a positive ex vivo drug sensitivity test.

We assessed the ex vivo sensitivity to venetoclax and bortezomib single agents and combination on the patient’s PBMCs and observed that the combination was beneficial (Fig. 5b, c). As a comparison, the sensitivities to two previously administered lines of venetoclax-based combinations – venetoclax + ibrutinib and venetoclax + acalabrutinib – were less potent, and these combinations did not show any additive effect on cell viability (Supplementary Fig. 6a). Following the drug sensitivity test, the patient received treatment with bortezomib in addition to venetoclax and ibrutinib, which were administered in the earlier line (Fig. 5a). In response to the treatment, levels of hemoglobin increased and leukocyte counts stabilized until week 12, which indicated an initial response to the treatment (Fig. 5d). Importantly, the patient had a significant improvement of quality of life and could return to a full-time job as a carpenter. However, the cancer progressed after 12 weeks on treatment (Fig. 5d). Levels of thrombocytes and granulocytes decreased as the disease progressed (Fig. 5e). The treatment was stopped after 18 weeks (Fig. 5a), and the patient later received treatment with venetoclax plus EPOCH (etoposide, prednisolone, vincristine, cyclophosphamide, doxorubicin) (Fig. 5a).

To study the impact of PI treatment on the immune cells and cell signaling, we performed combined immunophenotyping and (phospho)protein profiling on PBMCs collected at baseline, week 8, week 16, and off study (week 36). Figure 5f shows a representative t-SNE plot of the immunophenotype of the baseline sample. We observed marked changes in the immune cell composition during treatment (Fig. 5g–i). The proportion of CD19^+^ B cells increased, while the CD3^+^ T cell population and CD56^+^ NK cell population decreased (Fig. 5g). Furthermore, the proportion of CD8^+^ cytotoxic T cells decreased as the cancer relapsed (week 16), while the proportion of CD4^+^ T helper cells increased, along with regulatory T cells (Fig. 5h). Interestingly, we also observed that the proportion of CD56^bright^ NK cells increased after treatment stop, while the proportion of CD56^dim^ NK cells decreased (Fig. 5i). CD56^dim^ NK cells are more mature with greater cytotoxic abilities than CD56^bright^ NK cells, and their reduction at progression (week 36) may indicate reduced anti-tumor activity.

To investigate the impact of treatment response and resistance to bortezomib, we profiled the expression of 31 intracellular proteins in CD19^+^ B cells in the longitudinal samples (Fig. 5j, Supplementary Fig. 6b). In agreement with our observations in in vitro and ex vivo models, we found that in vivo treatment with a PI induced increased expression of Bim and Mcl-1 (Fig. 5k, left and middle). These levels were reduced upon progression (Fig. 5k, left and middle). Unlike the increase in Bcl-2 expression observed in CLL cells from treatment naïve patients ex vivo (Fig. 4b, center), we here observed a very slight decrease in Bcl-2 expression at week 8 compared to baseline, while there was a further reduction at progression (week 16), followed by an increase again off study (week 36) (Fig. 5k, right). This may suggest that Bcl-2 is regulated differently by proteasome inhibition in CLL cells from treatment naïve and heavily pre-treated patients.

## Discussion

Acquired resistance to targeted therapies is a general challenge in hemato-oncology. Since standard of care covers a limited number of therapeutic options, patients who are refractory to multiple lines of treatment generally have a poor prognosis. Continuous efforts are therefore invested in developing more effective, and hopefully curative, therapeutic strategies, as well as alternative therapies for treatment-refractory patients [5, 10]. PI3Ki are approved for hematologic malignancies, but have limited use since they are associated with severe toxicity [1]. ESMO recommends PI3Ki as late or last line therapy for CLL [4], and real-world data indicate clinical benefit from this strategy [7–9]. Since idelalisib is often used as a last resort, we aimed to identify treatment options for idelalisib-resistant B-cell malignancies. Therapies in development, including chimeric antigen receptor (CAR) T cells and bispecific antibodies, are not yet available to these patients outside of clinical trials. To identify more readily available treatment options, our strategy was therefore to perform a drug sensitivity screen with a drug library consisting of compounds that are either approved, under investigation, or have been shown to be effective ex vivo in hematologic malignancies. It was recently reported that the ERBB inhibitor lapatinib can recover sensitivity to idelalisib in the idelalisib-resistant KARPAS1718 cell line, although it did not have an effect as a single agent [14]. Unfortunately, we did not have ERBB inhibitors in our drug library, and we could therefore not study the effect of such agents in our system. Yet, we identified distinct functional phenotypes and actionable treatment sensitivities in the idelalisib-resistant B-cell malignancy models. The different resistance phenotypes we observed in KARPAS1718 and VL51 cells underscore the need for patient-tailored treatment strategies and add to the understanding of idelalisib-resistance mechanisms [11, 12].

Of interest, we found that ixazomib effectively reduced the viability of both idelalisib-resistant lymphoma models and primary CLL cells from patients who were either intolerant or resistant to idelalisib. Ixazomib is approved by EMA and FDA for treatment of MM, and it has demonstrated efficacy and tolerability at frontline in indolent B-cell non-Hodgkin lymphoma, alone and in combination with the CD20 antibody rituximab [40, 41]. Clinical trials with PIs have also been performed in CLL, but have shown limited treatment benefit [42, 43]. Notably, these studies were performed prior to the “era of targeted therapies”, so the place of PIs in the current treatment landscape remains undefined. However, it was recently shown that ixazomib is active in ibrutinib-resistant CLL [44], and we have previously treated an R/R CLL patient off-label with ixazomib following a positive drug sensitivity test [45]. Based on these encouraging reports, we are currently enrolling patients with R/R CLL to the clinical trial IMPRESS-Norway (NCT04817956). These patients will be treated with the first-in-class PI bortezomib if their CLL cells show ex vivo sensitivity to the drug. Here, we reported the outcome of the first patient enrolled in the CLL cohort of IMPRESS-Norway. Similar functional precision medicine strategies have previously been applied to other hematologic malignancies, mainly acute leukemias [46–48].

We found that the two idelalisib-resistant lymphoma models showed differential sensitivity to Bcl-2i, and Bcl-2i sensitivity correlated with Bcl-2 and Bim phosphorylation/expression. Levels of Bcl-2 family members have been reported to predict response to venetoclax in AML and MM [49, 50], while Bcl-2 expression did not correlate with overall response rate to venetoclax in a phase I study in non-Hodgkin lymphoma [51]. More recently, it was shown that hyperphosphorylation of Bcl-2 family members contributes to venetoclax resistance in lymphoid malignancies [34]. To validate our findings, it would be of interest to perform (phospho)protein profiling of a larger cohort of samples from patients with B-cell malignancies before and after development of resistance to Bcl-2i.

We found that treatment with a PI resulted in increased expression of Bcl-2 in some of our models, and combined treatment with a PI and venetoclax showed an additive effect on cell viability. It would therefore be of interest to also study the effects of combined treatment with a PI and an Mcl-1 inhibitor, since we observed that Mcl-1 expression increased in response to treatment with a PI across models.

Taken together, we have identified distinct phenotypic responses to idelalisib resistance in B-cell malignancy models and actionable treatment sensitivities. Proteasome inhibition was shown to have broad efficacy. Ongoing clinical investigations of PI as a salvage strategy in R/R CLL will provide additional insights into its clinical value in CLL.

## Supplementary information

Supplement

Supplementary Figures

Supplementary Table 1

Supplementary Table 2

Supplementary Table 3

Supplementary Table 4

Supplementary Table 5

## Data Availability

The data that support the findings of this study are available from the corresponding author (sigrid.skanland@ous-research.no) upon reasonable request.
