# Supplementary material for: Proteasome inhibition overcomes resistance to targeted therapies in B-cell malignancy models and in an index patient
Source: Cell Death Dis. 2025 Jul 23;16(1):555. doi: 10.1038/s41419-025-07884-7 (PMC12287370; doi:10.1038/s41419-025-07884-7)
Supplement: Supplementary file 1 — Supplement [file 41419_2025_7884_MOESM1_ESM.pdf]

SUPPLEMENT TO

Proteasome inhibition overcomes resistance to targeted therapies in B-cell malignancy models and in an index patient

Johanne U. Hermansen, Paschalis Athanasiadis, Yanping Yin, Anne-Sofie F. Rise, Alberto J. Arribas, Luciano Cascione, Hege G. Russnes, Åslaug Helland, Anthony R. Mato, Francesco Bertoni, Geir E. Tjønnfjord, Tero Aittokallio, Sigrid S. Skånland

This file contains:

Legends to Supplementary Figures 1-6

Legends to Supplementary Tables 1-5

References

SUPPLEMENTARY FIGURE LEGENDS

Supplementary Figure 1. Protein expression and Bcl-2 transcripts in KARPAS1718 and VL51 cell lines

a. Parental and idelalisib-resistant versions of KARPAS1718 (upper panel) and VL51 (lower panel) cell lines were fixed, permeabilized and stained with antibodies against the indicated (phospho)proteins. The cells were analyzed with a BD LSR Fortessa flow cytometer, and the data were analyzed with Cytobank (https://cellmass.cytobank.org/cytobank). Representative flow cytometry overlay histograms show raw counts of the indicated proteins. Par = parental cell line, res = resistant cell line.
b. The graph shows the RNA counts of the Bcl-2 transcripts in parental and idelalisib-resistant versions of KARPAS1718 and VL51 cell lines.

Supplementary Figure 2. Drug sensitivity screens with single agents and drug combinations in KARPAS1718 and VL51 cell lines

a. Parental (blue) and idelalisib-resistant (pink) KARPAS1718 (left graph) and VL51 (right graph) cell lines were treated with 93 single drugs at five different concentrations (1-10,000 nM) for 72h. Cell viability was assessed with the CellTiter-Glo luminescent cell viability assay. The graphs show the mean (bars) and individual (circles) drug sensitivity scores (DSS) to the different treatments for n=2-3 independent experiments for each cell line. The scores were calculated based on the area under the dose-response curves. High score indicates high sensitivity to the treatment.
b. KARPAS1718 and VL51 cell lines were treated with five different concentrations (1-10,000 nM) of idelalisib for 72h. Cell viability was assessed with the CellTiter-Glo luminescent cell viability assay. The graphs show the mean viability relative to the DMSO (0.1%) control of n=2 independent experiments with 6 technical replicates each per cell line. The error bars indicate standard deviation (SD).
c. As described in **a**, but the graphs show drug sensitivity scores for 87 drug combinations.

Supplementary Figure 3. RNA transcripts for proteasome subunits and proteasome the interactome

a. The heatmap shows the RNA counts (log_{10}) of the transcripts that belong to the proteasome group, as indicated in the HUGO Gene Nomenclature Committee database (HGNC) (1).

b. The heatmap shows the RNA counts (log_{10}) of the transcripts whose products interact with any of the proteasome subunit, as indicated in the HUGO Gene Nomenclature Committee database (HGNC) (1).

Supplementary Figure 4. Cell signaling changes induced by venetoclax and ixazomib treatments

a. Parental and idelalisib-resistant versions of KARPAS1718 and VL51 cell lines were treated with 50 nM venetoclax (V), ixazomib (I), or venetoclax + ixazomib (V+I) for 24h. The cells were stained with a fixable viability stain, fixed, barcoded and permeabilized, then stained with antibodies against intracellular proteins, as indicated. The cells were analyzed with a BD LSR Fortessa flow cytometer, and the data were analyzed with Cytobank (https://cellmass.cytobank.org/cytobank). Representative flow cytometry overlay histograms show raw counts of the indicated protein. Par = parental cell line, res = resistant cell line.

b. Experiment described in a**.** The signals are shown as noise corrected median fluorescence intensity (MFI) relative to the DMSO control which was set to zero (median of n=3).

c. Freshly thawed PBMCs from treatment naïve (n=3) CLL patients were treated with 1, 10, 25 or 50 nM venetoclax (V), ixazomib (I) or venetoclax + ixazomib (V+I) for 24h. The cells were stained with a fixable viability stain, fixed, barcoded and stained with anti-CD3 and anti-CD19 surface markers prior to permeabilization. The cells were next stained with antibodies against 34 intracellular proteins, as indicated. The samples were analyzed with a BD LSR Fortessa flow cytometer, and the data were analyzed with Cytobank (https://cellmass.cytobank.org/cytobank). The signal is shown as noise corrected MFI relative to the DMSO control which was set to zero (median of n=3) for $CD3^{-}CD19^{+}$ CLL cells.

Supplementary Figure 5. Primary CLL cells display reduced levels of Bcl-2 and reduced sensitivity to Bcl-2 inhibition.

a. Freshly thawed peripheral blood mononuclear cells (PBMCs) from treatment naïve (n=5) or idelalisib-resistant/intolerant (n=6) patients with CLL were fixed, permeabilized, and stained with the indicated antibodies. Signals were detected in $CD3^{-}CD19^{+}$ CLL by flow cytometry. Raw data were transformed to an arcsinh ratio relative to the signal of an isotype control, which was set to zero. The horizontal line indicates the median. Statistical testing was done with the Mann-Whitney test.

b. PBMCs from treatment naïve (n=7) or idelalisib-resistant/intolerant (n=9) patients with CLL were fixed were co-cultured with APRIL/BAFF/CD40L+ fibroblasts for 24h to prevent spontaneous apoptosis. The CLL cells were then separated from the fibroblast layer and treated with the indicated compounds at five different concentrations (1 nM – 10,000 nM) for 72h. Cell viability was assessed with the CellTiter-Glo luminescent cell viability assay. The graphs show the mean (bars) and individual (circles) drug sensitivity scores to the different treatments

Supplementary Figure 6. Ex vivo sensitivity to proteasome + BTK inhibitor combinations and flow cytometry data from a multi-resistant CLL index patient

a. Peripheral blood mononuclear cells (PBMCs) from the baseline sample of the multi-resistant CLL index patient were co-cultured with APRIL/BAFF/CD40L fibroblasts for 24h to prevent spontaneous apoptosis of the CLL cells. The CLL cells were then separated from the fibroblast layer and treated with venetoclax, acalabrutinib, ibrutinib, venetoclax + acalabrutinib, or venetoclax + ibrutinib at five different concentrations (1-10,000 nM) for 24h. Cell viability was assessed with the CellTiter-Glo luminescent cell viability assay and normalized to a negative control (0.1% DMSO). The graphs show the mean viability of triplicates, and the error bars indicate standard deviation (SD). Statistics were performed with a 2-way ANOVA with Bonferroni's multiple comparison correction when comparing single treatment to combination treatment and indicated for the comparison with venetoclax in grey

and for acalabrutinib or ibrutinib in orange. ns; not significant, ***p<0.001, ****p<0.0001.

b. PBMCs collected at baseline (B), week 8 (w8), week 16 (w16) after treatment start, and off study (OFS) were fixed, barcoded, stained with surface markers, then permeabilized and stained with antibodies against intracellular (phospho)proteins. The samples were analysed using a Cytek 5 L Aurora instrument, and the data were analyzed with Cytobank (https://cellmass.cytobank.org/cytobank). Representative flow cytometry overlay histograms showing raw count signals from $CD3^-CD19^+$ cells of the indicated protein for samples collected at the indicated time-points.

SUPPLEMENTARY TABLES

Supplementary Table 1. Compound library

Supplementary Table 2. Drug combinations

Supplementary Table 3. Patient characteristics

Supplementary Table 4. RNA counts. Due to the use of different filtering criteria, some of the transcripts previously identified (2,3) fell below the newly adopted thresholds. However, the directionality of the transcript levels between parental and resistant cell lines remained unchanged.

Supplementary Table 5. Annotation file

REFERENCES

1. Seal RL, Braschi B, Gray K, Jones TEM, Tweedie S, Haim-Vilmovsky L, et al. Genenames.org: the HGNC resources in 2023. Nucleic Acids Res. 2023 Jan;51(D1):D1003–9.
2. Arribas AJ, Napoli S, Cascione L, Sartori G, Barnabei L, Gaudio E, et al. Resistance to PI3κδ inhibitors in marginal zone lymphoma can be reverted by targeting the IL-6/PDGFRA axis. Haematologica. 2022 Apr;107(11):2685–97.
3. Arribas AJ, Napoli S, Cascione L, Barnabei L, Sartori G, Cannas E, et al. ERBB4-Mediated Signaling Is a Mediator of Resistance to PI3K and BTK Inhibitors in B-cell Lymphoid Neoplasms. Mol Cancer Ther. 2024 Mar;23(3):368–80.
