# Supplementary material for: Proteasome inhibition overcomes resistance to targeted therapies in B-cell malignancy models and in an index patient
Source: Cell Death Dis. 2025 Jul 23;16(1):555. doi: 10.1038/s41419-025-07884-7 (PMC12287370; doi:10.1038/s41419-025-07884-7)

Supplementary Figure 1

a.

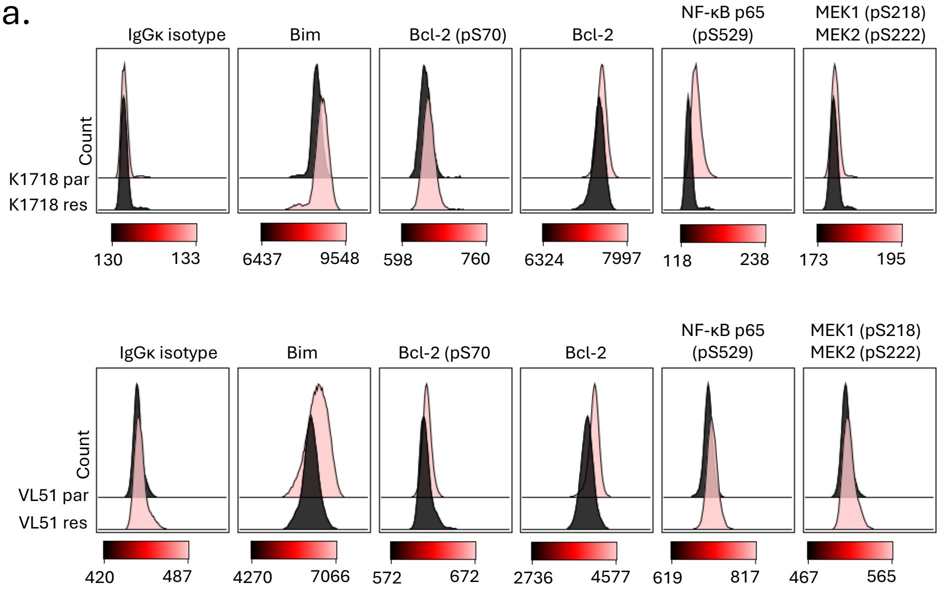

b.

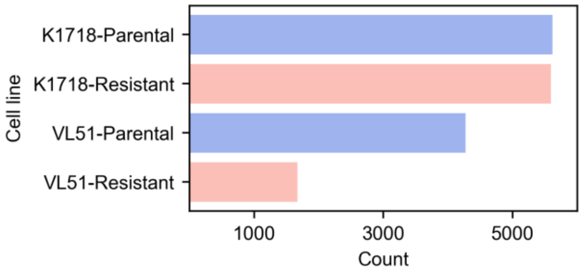

Supplementary Figure 2

a.

b.

c.

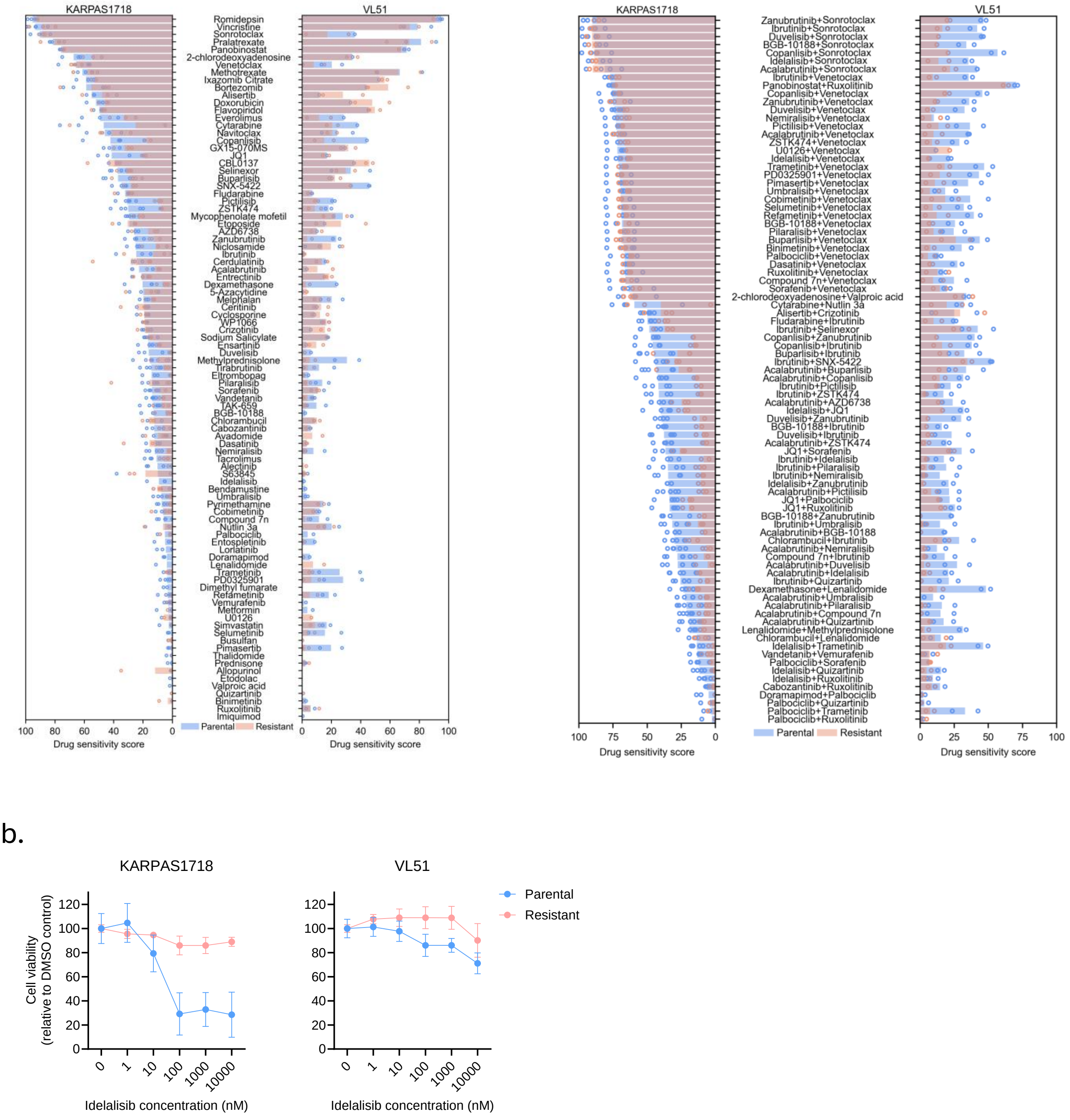

Supplementary Figure 3

a.

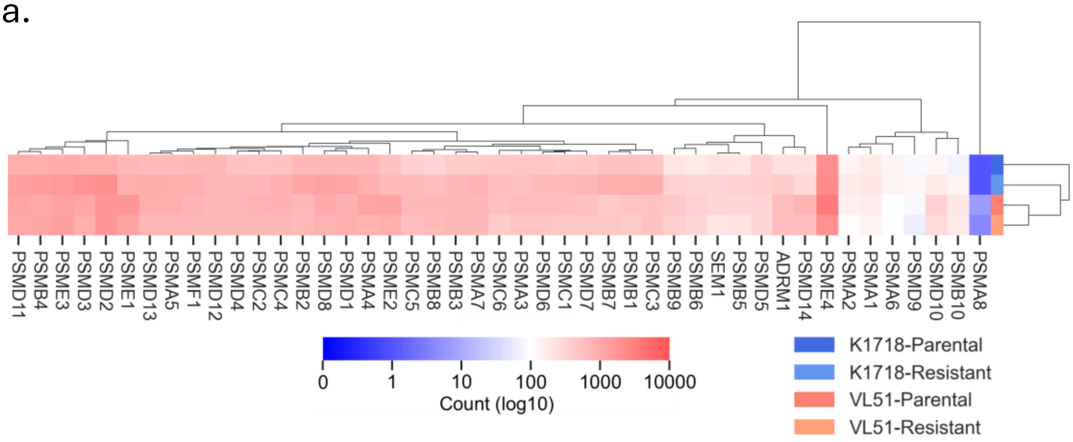

b.

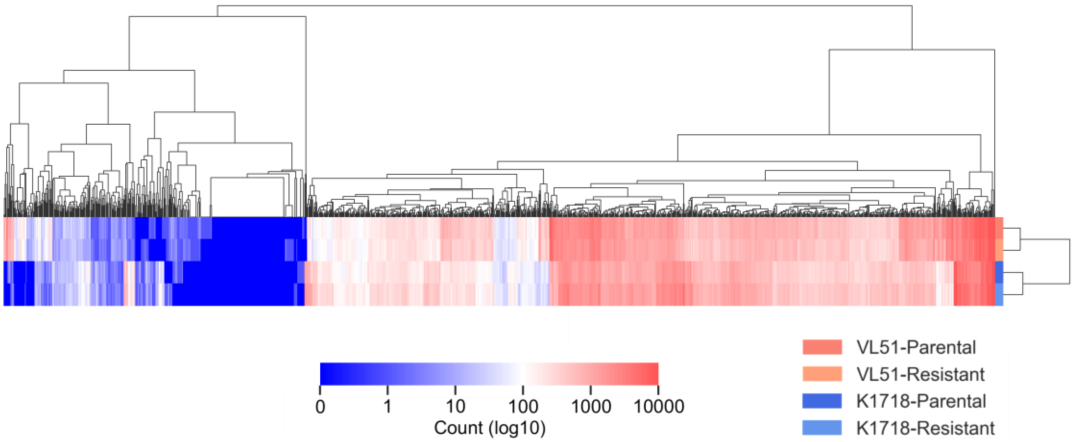

Supplementary Figure 4

a.

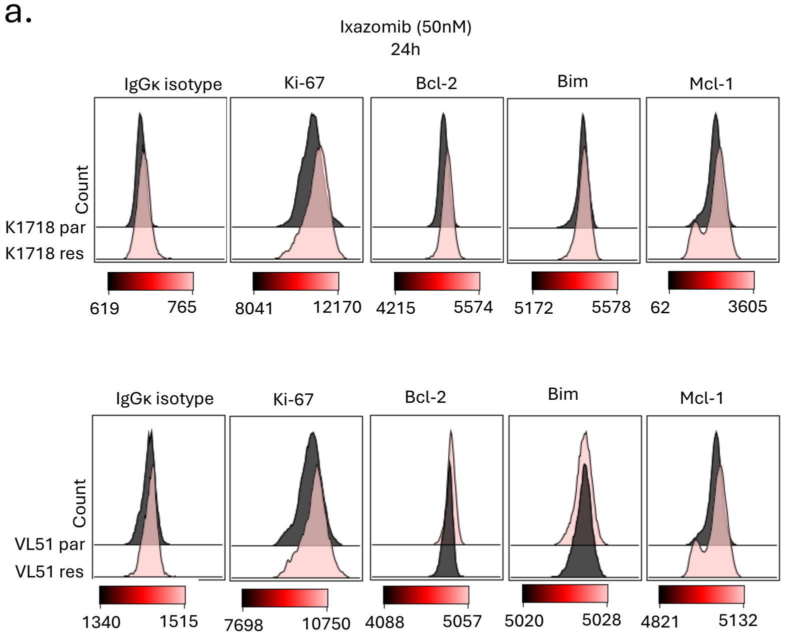

b.

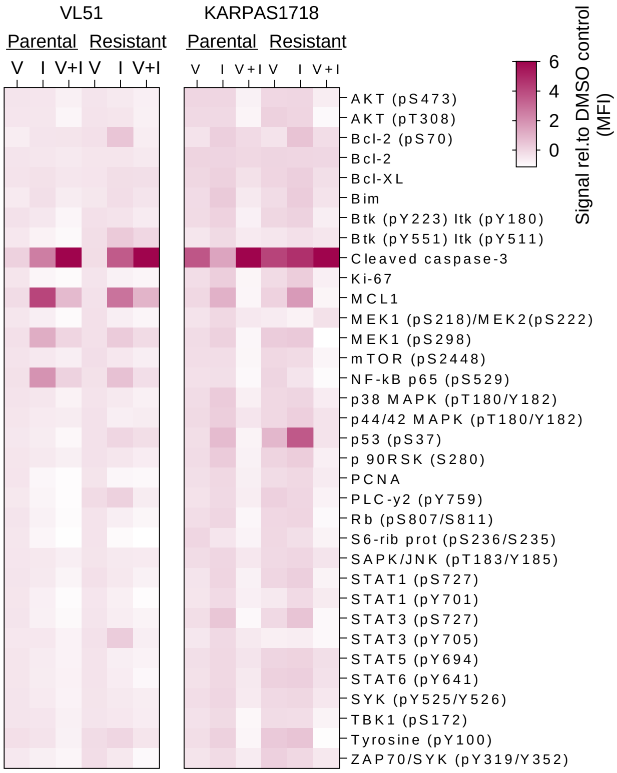

c.

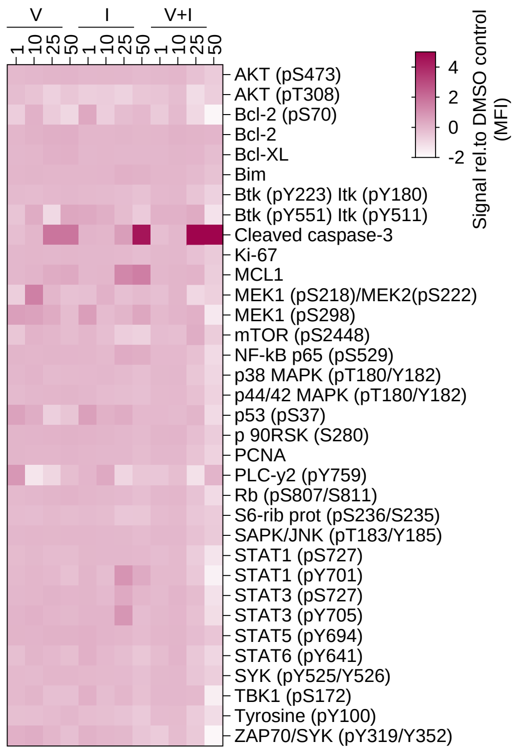

Supplementary Figure 5

a.

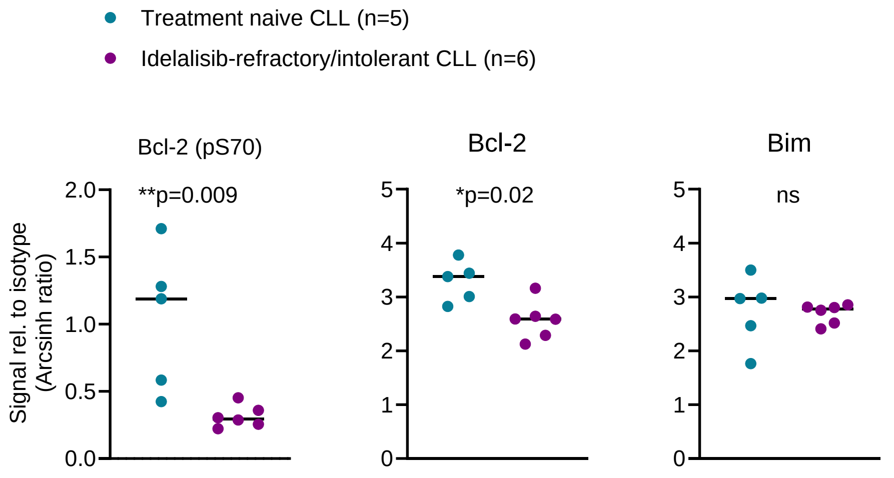

b.

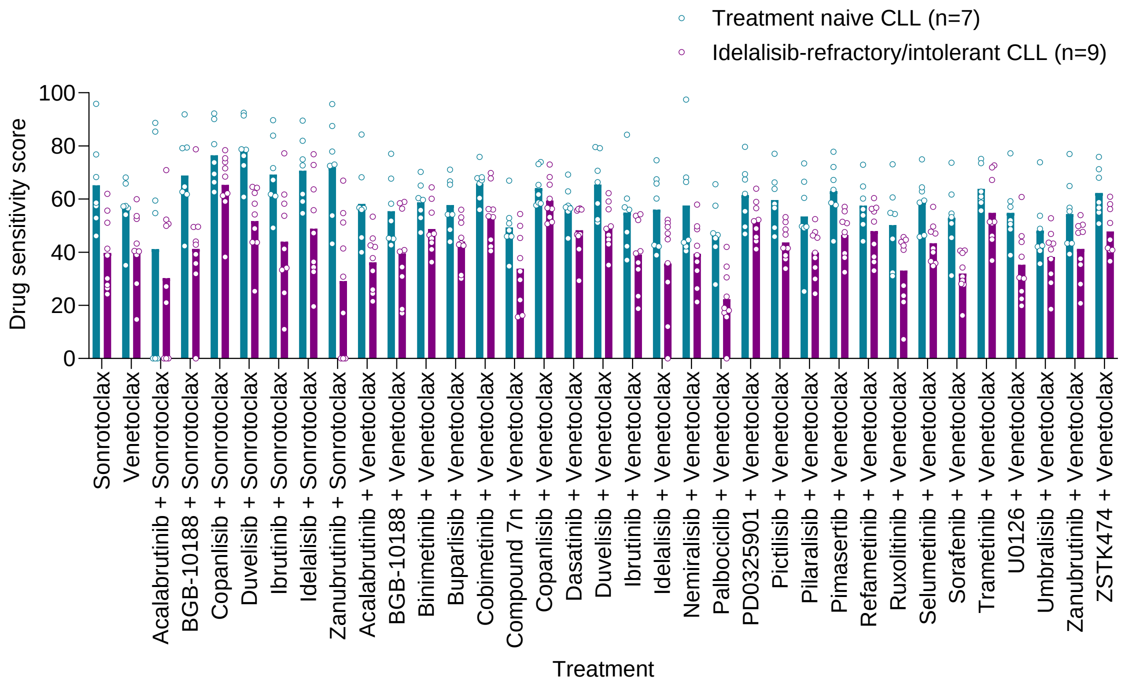

Supplementary Figure 6

a.

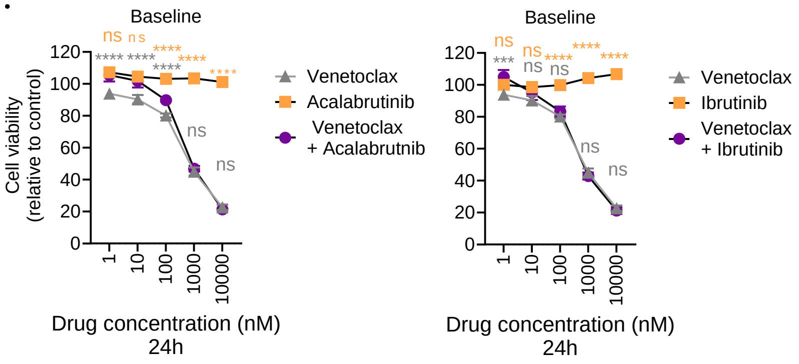

b.

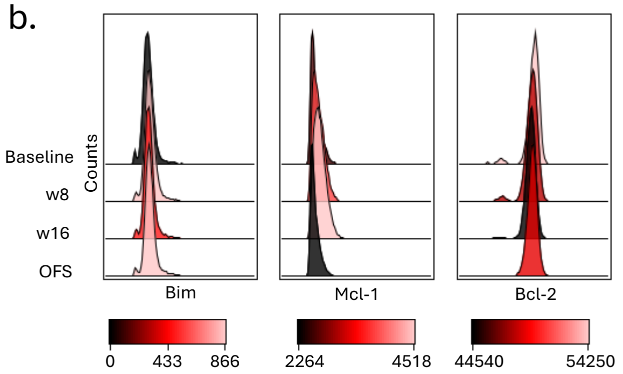

Supplement: Supplementary file 2 — Supplementary Figures [file 41419_2025_7884_MOESM2_ESM.pdf]
