# Supplementary material for: Proteasome inhibition overcomes resistance to targeted therapies in B-cell malignancy models and in an index patient
Source: Cell Death Dis. 2025 Jul 23;16(1):555. doi: 10.1038/s41419-025-07884-7 (PMC12287370; doi:10.1038/s41419-025-07884-7)
Supplement: Supplementary file 3 — Supplementary Table 1 [file 41419_2025_7884_MOESM3_ESM.docx]

**Supplementary Table 1.** Compound library

| **Compound** | **Target** | **Supplier** | **Cat no** | **Solvent** |
| --- | --- | --- | --- | --- |
| 2-chlorodeoxyadenosine | Adenosine deaminase | AK Scientific | E997 | DMSO |
| 5-Azacytidine | DNA methylation | AK Scientific | J10124 | DMSO |
| Acalabrutinib | BTK | Selleck Chemicals LLC | S8116 | DMSO |
| Alectinib | ALK | MedChemExpress | HY-13011 | DMSO |
| Alisertib | Aurora A | Selleck Chemicals LLC | S1133 | DMSO |
| Allopurinol | Xanthine oxidase | ENAMINE Ltd. | Z228474686 | DMSO |
| Avadomide | Cereblon | TargetMol | T3549 | DMSO |
| AZD6738 | ATR | TargetMol | T3338 | DMSO |
| Bendamustine | DNA-damaging | Selleck Chemicals LLC | S1212 | DMSO |
| BGB-10188 | PI3K | BeiGene |  | DMOS |
| Binimetinib | MEK1/2 | Selleck Chemicals LLC | S7007 | DMSO |
| Bortezomib | 20S proteasome | Selleck Chemicals LLC | S1013 | DMSO |
| Buparlisib | PI3K | Selleck Chemicals LLC | S2247 | DMSO |
| Busulfan | Alkylating agent | ENAMINE Ltd. | Z276508890 | DMSO |
| Cabozantinib | VEGFR2 | LC Laboratories | C-8901 | DMSO |
| CBL0137 | p53/NF-kB | TargetMol | T4126 | DMSO |
| Cerdulatinib | JAK1/2/3, TYK2, SYK | TargetMol | T6104 | DMSO |
| Ceritinib | ALK | MedChemExpress | HY-15656 | DMSO |
| Chlorambucil | Alkylating agent | Pure Chemistry Scientific | 815818 | DMSO |
| Cobimetinib | MEK1/2 | Selleck Chemicals LLC | S8041 | DMSO |
| Compound 7n | PI3K | Selleck Chemicals LLC | S8693 | DMSO |
| Copanlisib | PI3K | Selleck Chemicals LLC | S2802 | 5% TFA |
| Crizotinib | c-Met, ALK | LC Laboratories | C-7900 | DMSO |
| Cyclosporine | Calcineurin phosphatase | TargetMol | T6459 | DMSO |
| Cytarabine | DNA synthesis | Selleck Chemicals LLC | S1648 | H2O |
| Dasatinib | Abl, Src, c-Kit | LC Laboratories | D-3307 | DMSO |
| Dexamethasone | Interleukin receptor | Selleck Chemicals LLC | S1322 | DMSO |
| Dimethyl fumarate | Unknown | Vitas-M Laboratory, Ltd. | STK039379 | DMSO |
| Doramapimod | p38 MAPK | LC Laboratories | D-2744 | DMSO |
| Doxorubicin | DNA topoisomerase II | Selleck Chemicals LLC | S1208 | DMSO |
| Duvelisib | PI3K | Selleck Chemicals LLC | S7028 | DMSO |
| Eltrombopag | Thrombopoietin receptor | Cayman Europe | 13247.0 | DMSO |
| Ensartinib | ALK | Selleck Chemicals LLC | S2934 | DMSO |
| Entospletinib | SYK | Cayman Europe | 17653.0 | DMSO |
| Entrectinib | ALK | Selleck Chemicals LLC | S7998 | DMSO |
| Etodolac | COX | Selleck Chemicals LLC | S1328 | DMSO |
| Etoposide | Topoisomerase II | Cayman Europe | 12092.0 | DMSO |
| Everolimus | mTOR | Selleck Chemicals LLC | S1120 | DMSO |
| Flavopiridol | CDK1/2/4/6 | TargetMol | T2615 | DMSO |
| Fludarabine | DNA synthesis, STAT1 | Selleck Chemicals LLC | S1491 | DMSO |
| GX15-070MS (Obatoclax) | Bcl-2 | LC Laboratories | S2680 | DMSO |
| Ibrutinib | BTK | LC Laboratories | S2680 | DMSO |
| Idelalisib | PI3K | LC Laboratories | S2226 | DMSO |
| Imiquimod | Immune response | Vitas-M Laboratory, Ltd | STK583860 | DMSO |
| Ixazomib Citrate | 20S proteasome | Cayman Europe | 18386.0 | DMSO |
| JQ1 | BET bromodomain | Selleck Chemicals LLC | S7110 | DMSO |
| Lenalidomide | Immunomodulation | Selleck Chemicals LLC | S1029 | DMSO |
| Lorlatinib | ALK | Selleck Chemicals LLC | Lorlatinib | DMSO |
| Melphalan | Antineoplastic activity | AK Scientific | H877 | DMSO |
| Metformin | Glucose production | Vitas-M Laboratory, Ltd. | STK011633 | DMSO |
| Methotrexate | Dihydrofolate reductase (DHFR) | Selleck Chemicals LLC | S1210 | DMSO |
| Methylprednisolone | Glucocorticoid receptor | Selleck Chemicals LLC | S1733 | DMSO |
| Mycophenolate mofetil | Inosine monophosphate dehydrogenase I/II | AK Scientific | H944 | DMSO |
| Navitoclax | Bcl-xL, Bcl-2 and Bcl-w | Selleck Chemicals LLC | S1001 | DMSO |
| Nemiralisib | PI3K | Selleck Chemicals LLC | S7937 | DMSO |
| Niclosamide | DNA replication, STAT3 | Selleck Chemicals LLC | S3030 | DMSO |
| Nutlin 3a | p53/MDM2 | Selleck Chemicals LLC | S8059 | DMSO |
| Palbociclib | CDK4/6 | LC Laboratories | P-7744 | DMSO |
| Panobinostat | HDAC | LC Laboratories | P-3703 | DMSO |
| PD0325901 | MEK1/2 | Selleck Chemicals LLC | S1036 | DMSO |
| Pictilisib | PI3K | Selleck Chemicals LLC | S1065 | DMSO |
| Pilaralisib | PI3K | Selleck Chemicals LLC | S7645 | DMSO |
| Pimasertib | MEK1/2 | Selleck Chemicals LLC | S1475 | DMSO |
| Pralatrexate | Antifolate | TargetMol | T6120 | DMSO |
| Prednisone | Immunosuppressant | TargetMol | T1018 | DMSO |
| Pyrimethamine | Dihydrofolate reductase (DHFR) | AK Scientific | J10587 | DMSO |
| Quizartinib | FLT3 | LC Laboratories | Q-4747 | DMSO |
| Refametinib | MEK1/2 | Selleck Chemicals LLC | S1089 | DMSO |
| Romidepsin | HDAC1/2 | Selleck Chemicals LLC | S3020 | DMSO |
| Ruxolitinib | JAK1/2 | LC Laboratories | R-6600 | DMSO |
| S63845 | Mcl-1 | Selleck Chemicals LLC | S8383 | DMSO |
| Selinexor | CRM1 | Cayman Europe | 18127.0 | DMSO |
| Selumetinib | MEK1/2 | Selleck Chemicals LLC | S1008 | DMSO |
| Simvastatin | HMG-CoA reductase | Vitas-M Laboratory, Ltd. | STK801938 | DMSO |
| SNX-5422 | HSP90 | Cayman Europe | 18270.0 | DMSO |
| Sodium Salicylate | Anti-inflammatory | SantaCruz | sc-3520 | H2O |
| Sonrotoclax | Bcl-2 | BeiGene |  | DMSO |
| Sorafenib | Raf-1, B-Raf and VEGFR-2 | LC Laboratories | S-8599 | DMSO |
| Tacrolimus | FKBP12 (FK506 binding protein) | AK Scientific | G448 | DMSO |
| TAK-659 | SYK | TargetMol | T4209 | DMSO |
| Thalidomide | Immunomodulation, E3 ubiquitin ligase | Vitas-M Laboratory, Ltd. (Premium) | STL356025 | DMSO |
| Tirabrutinib | BTK | Ark Pharm, Inc. | AK547263 | DMSO |
| Trametinib | MEK1/2 | LC Laboratories | T-8123 | DMSO |
| U0126 | MEK1/2 | Selleck Chemicals LLC | S1102 | DMSO |
| Umbralisib | PI3K | MedChemTronica | HY-12279 | DMSO |
| Valproic acid | Histone deacetylase | ENAMINE Ltd. | Z1511532065 | DMSO |
| Vandetanib | VEGFR2/3, EGFR | LC Laboratories | V-9402 | DMSO |
| Vemurafenib | B-Raf^V600E^ | LC Laboratories | V-2800 | DMSO |
| Venetoclax | Bcl-2 | Selleck Chemicals LLC | S8048-5MG | DMSO |
| Vincristine | Microtubules | Selleck Chemicals LLC | S1241 | DMSO |
| WP1066 | JAK2, STAT3 | Selleck Chemicals LLC | S2796 | DMSO |
| Zanubrutinib | BTK | BeiGene |  | DMSO |
| ZSTK474 | PI3K | Selleck Chemicals | S1072 | DMSO |
