# Supplementary material for: Proteasome inhibition overcomes resistance to targeted therapies in B-cell malignancy models and in an index patient
Source: Cell Death Dis. 2025 Jul 23;16(1):555. doi: 10.1038/s41419-025-07884-7 (PMC12287370; doi:10.1038/s41419-025-07884-7)
Supplement: Supplementary file 4 — Supplementary Table 2 [file 41419_2025_7884_MOESM4_ESM.docx]

**Supplementary table 2.** Drug combinations

| **Drug 1** | **Drug 2** |
| --- | --- |
| 2-chlorodeoxyadenosine | Valproic acid |
| Acalabrutinib | AZD6738 |
| Acalabrutinib | BGB-10188 |
| Acalabrutinib | Sonrotoclax |
| Acalabrutinib | Buparlisib |
| Acalabrutinib | Compound 7n |
| Acalabrutinib | Copanlisib |
| Acalabrutinib | Duvelisib |
| Acalabrutinib | Idelalisib |
| Acalabrutinib | Nemiralisib |
| Acalabrutinib | Pictilisib |
| Acalabrutinib | Pilaralisib |
| Acalabrutinib | Quizartinib |
| Acalabrutinib | Umbralisib |
| Acalabrutinib | Venetoclax |
| Acalabrutinib | ZSTK474 |
| Alisertib | Crizotinib |
| BGB-10188 | Sonrotoclax |
| BGB-10188 | Ibrutinib |
| BGB-10188 | Venetoclax |
| BGB-10188 | Zanubrutinib |
| Binimetinib | Venetoclax |
| Buparlisib | Ibrutinib |
| Buparlisib | Venetoclax |
| Cabozantinib | Ruxolitinib |
| Chlorambucil | Ibrutinib |
| Chlorambucil | Lenalidomide |
| Cobimetinib | Venetoclax |
| Compound 7n | Ibrutinib |
| Compound 7n | Venetoclax |
| Copanlisib | Ibrutinib |
| Copanlisib | Venetoclax |
| Copanlisib | Zanubrutinib |
| Cytarabine | Nutlin 3a |
| Dasatinib | Venetoclax |
| Dexamethasone | Lenalidomide |
| Doramapimod | Palbociclib |
| Duvelisib | Ibrutinib |
| Duvelisib | Venetoclax |
| Duvelisib | Zanubrutinib |
| Fludarabine | Ibrutinib |
| Ibrutinib | Idelalisib |
| Ibrutinib | Nemiralisib |
| Ibrutinib | Pictilisib |
| Ibrutinib | Pilaralisib |
| Ibrutinib | Quizartinib |
| Ibrutinib | Selinexor |
| Ibrutinib | SNX-5422 |
| Ibrutinib | Umbralisib |
| Ibrutinib | Venetoclax |
| Ibrutinib | ZSTK474 |
| Idelalisib | JQ1 |
| Idelalisib | Quizartinib |
| Idelalisib | Ruxolitinib |
| Idelalisib | Trametinib |
| Idelalisib | Venetoclax |
| Idelalisib | Zanubrutinib |
| JQ1 | Palbociclib |
| JQ1 | Ruxolitinib |
| JQ1 | Sorafenib |
| Lenalidomide | Methylprednisolone |
| Nemiralisib | Venetoclax |
| Palbociclib | Quizartinib |
| Palbociclib | Ruxolitinib |
| Palbociclib | Sorafenib |
| Palbociclib | Trametinib |
| Palbociclib | Venetoclax |
| Panobinostat | Ruxolitinib |
| PD0325901 | Venetoclax |
| Pictilisib | Venetoclax |
| Pilaralisib | Venetoclax |
| Pimasertib | Venetoclax |
| Refametinib | Venetoclax |
| Ruxolitinib | Venetoclax |
| Selumetinib | Venetoclax |
| Sonrotoclax | Copanlisib |
| Sonrotoclax | Duvelisib |
| Sonrotoclax | Ibrutinib |
| Sonrotoclax | Idelalisib |
| Sonrotoclax | Zanubrutinib |
| Sorafenib | Venetoclax |
| Trametinib | Venetoclax |
| U0126-EtOH | Venetoclax |
| Umbralisib | Venetoclax |
| Vandetanib | Vemurafenib |
| Venetoclax | Zanubrutinib |
| Venetoclax | ZSTK474 |
