# Supplementary material for: Proteasome inhibition overcomes resistance to targeted therapies in B-cell malignancy models and in an index patient
Source: Cell Death Dis. 2025 Jul 23;16(1):555. doi: 10.1038/s41419-025-07884-7 (PMC12287370; doi:10.1038/s41419-025-07884-7)
Supplement: Supplementary file 5 — Supplementary Table 3 [file 41419_2025_7884_MOESM5_ESM.docx]

**Supplementary Table 3.** Patient characteristics

| **Patient ID** | **Gender** | **Age** | **IGVH** | **FISH/TP53** | **Treatment at procurement** | **Treatment prior to procurement** | **Treatment status as indicated in figures** |
| --- | --- | --- | --- | --- | --- | --- | --- |
| CLL002D | m | 66 | U | n.e. | None | None | Treatment naive |
| CLL003D | m | 73 | M | n.e. | None | None | Treatment naive |
| CLL103 | m | 62 | U | del(13q14) | Idelalisib | FCR, idelalisib | Idelalisib intolerant |
| CLL116 | m | 82 | M | n.e. | None | Radiation therapy for prostate cancer | Treatment naive |
| CLL163 | f | 66 | M | n.e. | None | None | Treatment naive |
| CLL164 T1 | m | 73 | U | del(13q14), TP53 mutation | None | Ibrutinib, idelalisib | Idelalisib intolerant |
| CLL177 | f | 69 | M | n.e. | None | None | Treatment naive |
| CLL183 | m | 68 | M | Normal | None | FC, venetoclax, idelalisib | Idelalisib intolerant |
| CLL187 | m | 66 | M | No NOTCH1, SF3B1 or TP53 mutation | None | None | Treatment naive |
| CLL200 | m | 64 | U | del(13q14), del(17p)  TP53 mutation, SF3B1 mutation, and NRA mutation | None | Idelalisib | Idelalisib intolerant |
| CLL206 | f | 66 | U | 46,XX, heterozygous del(13q14) | None | None | Treatment naive |
| CLL211 | m | 80 | U | trisomy 12, del(11q22) | None | BR, idelalisib | Idelalisib refractory |
| CLL248 | m | 73 | M | del(13q14) + translocation involving 14q32 | None | None | Treatment naive |
| CLL249 | f | 80 | n.e. | n.e. | None | None | Treatment naive |
| CLL254 | m | 32 | U | Normal karyotype; NOTCH1-mutation | None | None | Treatment naive |
| CLL257 | m | 75 | M | Normal karyotype | None | None | Treatment naive |
| IMPR.C-0001 | m | 60 | UM | *46,XY,t(13;20)(q13;q13) and hemizygous* *del(17)(p13)*  TP53 mutation, Arg175His mutation, BTK Cys481Ser mutation, Lys601Gly mutation, SF3B1 mutation, PTEN Phe278Leu mutation | See figure 5a | FCR, ibrutinib, venetoclax + R, venetcolax + Obinutuzumab, venetoclax + ibrutinib, venetoclax + acalabrutinib, idelalisib, venetoclax + ibrutinib + bortezomib, venetoclax + EPOCH |  |
| UMB2 HPA-0205-screening | m | 82 | U | Failed | None | BR, Rx2, Rx1 Cytoxan x2, ibrutinib, idelalisib | Idelalisib intolerant |
| UMB3 HPA-0206-screening | m | 72 | M | del(11q22), TP53 mutation | None | FRx4, FCRx6, BR x5, ibrutinib, idelalisib + R, obinutuzumab | Idelalisib intolerant |
| UMB5 NYA-0204-C2D1 | f | 78 | M | Normal | Umbralisib | Idelalisib | Idelalisib intolerant |
| UMB16 FMB-0202-screening | m | 80 | M | Normal | None | Chl, Rx2, CVP, BR, idelalisib | Idelalisib intolerant |
| UMB49  DCA-0201  screening | m | 59 | n.e | n.e | None | FR, lenalidomid, OFA + B, idelalisib, R + idelalisib | Idelalisib intolerant |
| UMB55  PHA-0206  screening | m | 57 | n.e | n.e | None | FCR, R, BR, ibrutinib x2, lenalidomide, venetoclax, idelalisib, obinutuzumab | Idelalisib intolerant |
| UMB75  PHA-0217  Screening | f | 78 | n.e. | n.e | None | BR, ibrutinib, idelalisib + R | Idelalisib intolerant |
| UMB104  NYA-0204  screening | f | 78 | M | n.e | None | Idelalisib | Idelalisib intolerant |

B, bendamustine; BTK, bruton tyrosine kinase; C, cyclophosphamide; Chl; chlorambucil; EPOCH, etoposide, prednisolone, vincristine, cyclophosphamide, doxorubicin; f, female; F, fludarabine; m, male; M, IGVH mutated CLL; n.e., not established; OFA, ofatumumab; P, prednisone; R, rituximab; U, IGVH unmutated CLL; V, vincristine;
